# Room temperature, metal-free, CDI-promoted, ex-situ protocol for S-methyl thioester synthesis

**DOI:** 10.1038/s41598-025-97728-3

**Published:** 2025-05-27

**Authors:** Manisha A. Patel, Vandana Sharma, Rupali Chavan, Juhi Pal, Santosh J. Gharpure, Anant R. Kapdi

**Affiliations:** 1 Department of Chemistry, Institute of Chemical Technology, Nathalal Parekh Road, Matunga, Mumbai, 400019 India; 2 Department of Chemistry, Indian Institute of Technology Bombay, Powai, Mumbai, 400076 India

**Keywords:** Chemical biology, Chemistry

## Abstract

S-methyl thioesters have proven to be useful intermediates in many biosynthetic reactions and their occurrence in bioactive molecules further enhance their appeal. Shortcomings in several reported synthetic protocols have led us to disclose herewith a mild, room temperature ex situ protocol that provides access to a large number of thioesters (aryl, heteroaryl, alkyl, amino acids). Late-stage functionalization strategies were also performed on commercial pharmaceutical drugs (containing carboxylic acid functionality) while further in situ conversion to valuable compounds has also been achieved.

**Supplementary Information:**

The online version contains supplementary material available at 10.1038/s41598-025-97728-3.

## Introduction

**Fig. 1:**
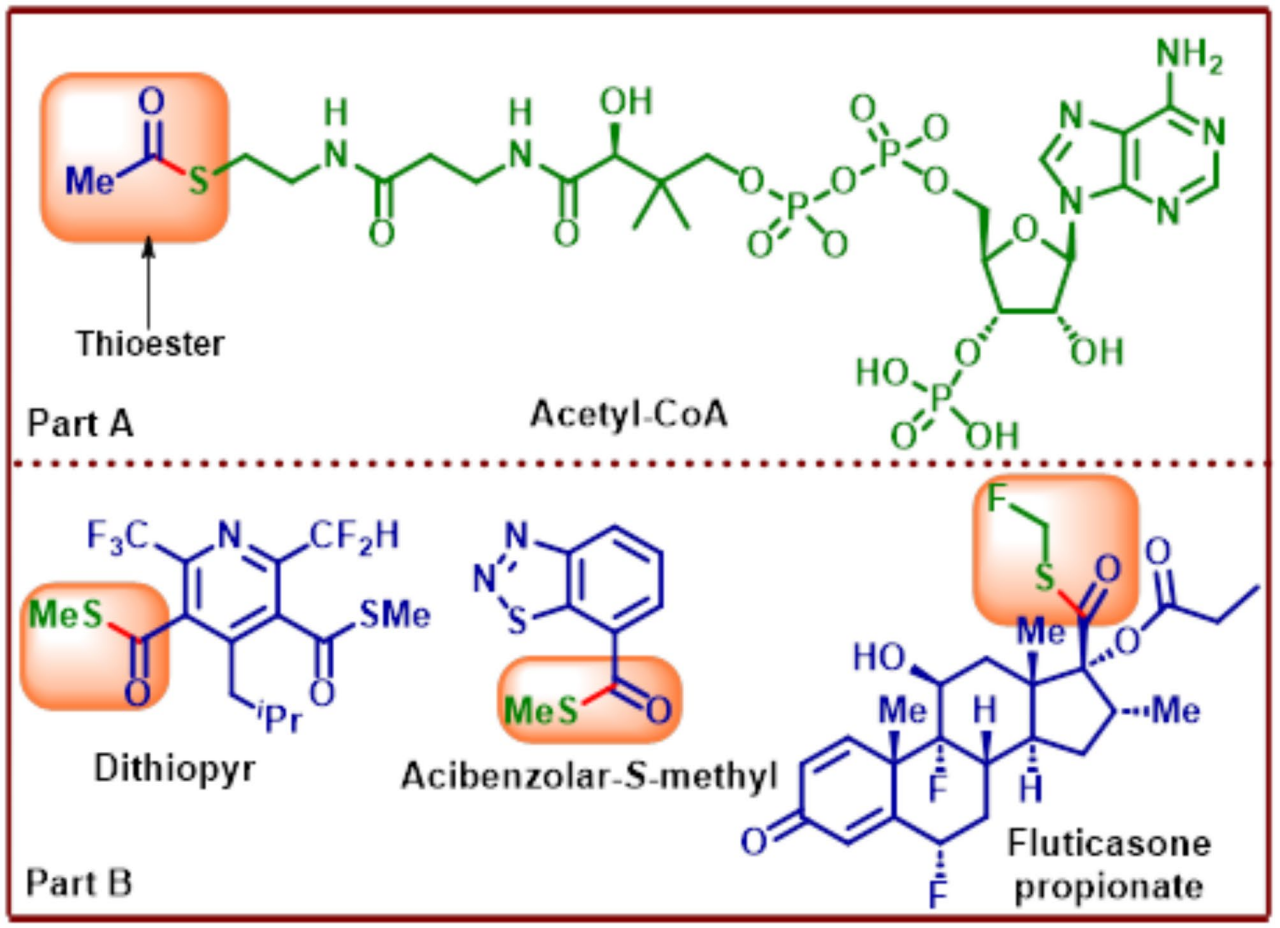
Part (**A**) Thioester functionality in acetyl coenzyme-A. Part (**B**) Thioester containing bio-active and drug molecules.

The ubiquitous nature of acetyl coenzyme A (Acetyl-CoA) in many biochemical reactions as a molecule associated with the delivery of acetyl group (in Krebs cycle) is probably the most striking example of alkyl thioesters that have proven to be key intermediates capable of converting into useful compounds readily by undergoing simple transformations (Fig. 1A)^1,2^.

The significant presence of thioesters in biology as the possible precursors of life leads to their involvement in key biosynthetic reactions such as fatty acids, mevalonate synthesis and utilization (for steroid biosynthesis), synthesis of various peptides, terpenes, porphyrins, lipids in the body, tagging of proteins with ubiquitin etc^3–7^. further justifies the focus given to them by scientists. Recognition of thioester as a reactive functionality and its utilization in the bioconjugation of proteins increased significantly after Dawson first reported a simple chemical ligation methodology in 1994^8^. Given the importance of these useful intermediates and their vast applications in fields ranging from synthesis, chemical ligation, intein splicing, bioconjugation has provided synthetic chemists with an opportunity to explore milder and expedient methodologies for obtaining these structural motifs. S-Methyl thioester amongst others is the simplest but has found extensive usage in many of the processes discussed above besides its occurrence in naturally occurring bio-active molecules as well as pharmaceutical drugs (**B**)^8–12^. The traditional routes for the synthesis of S-methyl thioesters involving the reaction between carboxylic acid derivatives and methanethiol (or the corresponding salt) have been plagued with several problems such as poor yields and harsh reaction conditions^13^. Difficulty in handling the gaseous methanethiol (pressurized gas cylinders) is the primary reason for researchers to explore methodologies that employ precursors capable of producing methanethiol in the laboratory set up.

Transition-metal catalyzed conversion of carboxylic acids and aryl halides into *S*-methyl thioesters was recently disclosed using dimethyldisulfide or *S*-methylalkyl thioether as the methanethiol precursor in the presence of Ni catalyst at ambient temperature and Pd-catalyst at temperature > 100 °C, respectively (**a**)^14,15^. Photocatalytic conversion of carboxylic acid to S-methyl thioester has also been disclosed using dimethyldisulfide under nickel catalysis with an iridium-based photocatalyst. Authors report an extensive substrate scope but the requirement of two precious and expensive metal catalysts makes it less attractive for further applications (**b**)^16^. A metal-free version involving the use of DMSO as the reaction solvent as well as the methanethiol precursor at 180 °C has also been described in the literature, however, with serious limitations pertaining to poor reactivity, limited scope and hazardous conditions (**c**)^17^.

To circumvent all these problems, we disclose herein a simple, ambient temperature, metal-free protocol involving the reaction of a variety of in situ activated carboxylic acids [by reaction with carbonyl diimidazole (CDI)] with the ex situ generated methanethiol gas in good to excellent yields. The protocol provides easy access to a wide number of S-methyl thioesters of aromatic/aliphatic/heteroaromatic carboxylic acids as well as α-amino acids. The mildness of the method is demonstrated in late-stage functionalization of important commercial pharmaceutical drugs containing carboxylic acid functionality to the corresponding *S*-methyl thioesters.

**Fig. 2:**
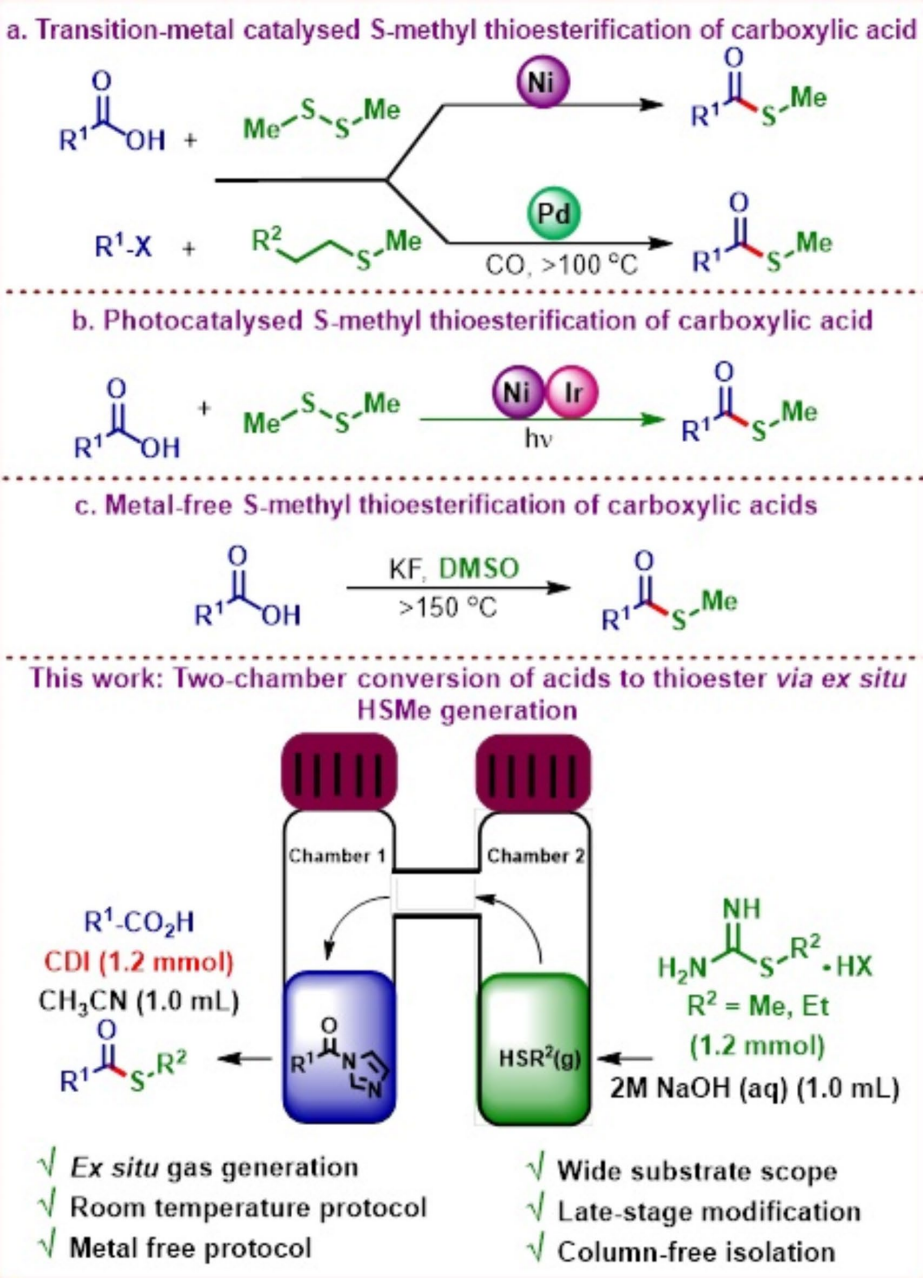
Strategies for *S*-methyl thioesterification of carboxylic acid in literature.

S-Methyl thioesters are important synthetic functionalities sometimes performing the crucial role of being an intermediate in several synthetic transformations for obtaining aldehydes, ketones, amides etc. Previously reported methods for S-methyl thioester synthesis have been discussed in the previous section with each exhibiting some limitation while a possible alternative to these could involve a metal-free activation of carboxylic acid using coupling reagents. Reagents such as dicyclohexylcarbodiimide (DCC), *N,N,N*′,*N*′-tetramethyl-*O*-(*N*-succinimidyl)uronium tetrafluoroborate (TSTU), benzotriazol-1-yloxytris(dimethylamino)phosphonium hexafluorophosphate (BOP), hexafluorophosphate azabenzotriazole tetramethyl uronium (HATU), *N*-hydrosuccinimide (NHS), carbonyldiimidazole (CDI) have been known to form reactive intermediates with carboxylic acids that can rapidly undergo reactions with a wide variety of nucleophiles finding applications especially in the area of bio-conjugation of various bioactive molecules with proteins^18–22^. However, from a cost and reactivity point of view, NHS and CDI are preferred. These in situ formed intermediates could further undergo reaction with thiomethyl nucleophile (either as gaseous methanethiol or as thiomethyl anion formed via one of the previously described methods). The possibility of using toxic and acrid smelling MeSH gas using a pressurized cylinder is not a feasible option and to circumvent this problem, surrogate molecules that can locally generate MeSH with a simple reaction with a reagent such as NaOH could provide a safer alternative for obtaining *S-*methyl thioesters.

## Results and discussion

A wide variety of gaseous nucleophiles or reactants such as HNMe_2_, MeSH, H_2_, CO, CO_2_, NH_3_ etc. are synthetically accessed using their surrogates^23–25^. The localized formation and in situ consumption of these gaseous reactants minimizes the hazards involved with the pressurized systems, which if leaked, could lead to fatal consequences in some of these cases. It is also worth noting that the reactive intermediates formed by the action of NHS or CDI on carboxylic acid are sensitive to moisture and are known to hydrolyze rapidly and therefore over the years, researchers have devised equipment that can separate the operations involving the generation of gaseous nucleophiles (water might be needed as part of the process) from the reactive intermediates. A two-chamber reaction set-up has been devised by several researchers e.g. H-tube by Skydstrup^26^, Lambda-tube by Ananikov^27^, in-ex reactor by Wu^28^ for performing these transformations. In the past few years, our research group has also worked extensively on the generation of gaseous nucleophiles using cheap and easily available reagents, and under metal-catalyzed as well as metal-free conditions at ambient temperature for the modification of several substrates including heteroarenes and carboxylic acids^29–31^. Similar to the reactors described previously, our research group also developed a H-tube two-chamber apparatus for handling of these gaseous reactants. Modification of chloroheteroarenes was undertaken by generating HNMe_2_ gas (DMF was used as a surrogate in the presence of a strong base such as KO*t*Bu) in an in situ manner at room temperature and using Cu/PTABS (KapdiPhos) catalytic system to furnish *N*,*N*-dimethylamino-functionalized heteroarenes. The methodology was further extended towards the synthesis of *N*,*N*-dimethylamido-functionalized arenes and heteroarenes first time via an ex situ approach, wherein a reactive intermediate was generated from the reaction of (hetero)aryl carboxylic acids and CDI in one of the chambers and subsequent reaction with an ex situ generated HNMe_2_ gas (second chamber). The idea for the synthesis of *S-*methyl thioesters stem from these previous two methodologies but for the execution, we need to identify a suitable surrogate that will be capable of generating MeSH.

In recent years, *S-*methylisothiourea hemisulfate has been one of the most widely used, inexpensive, readily available and odorless surrogate first introduced by Skydstrup for the ex situ generation of MeSH by the action of a strong base like NaOH^32^.

**Fig. 3:**
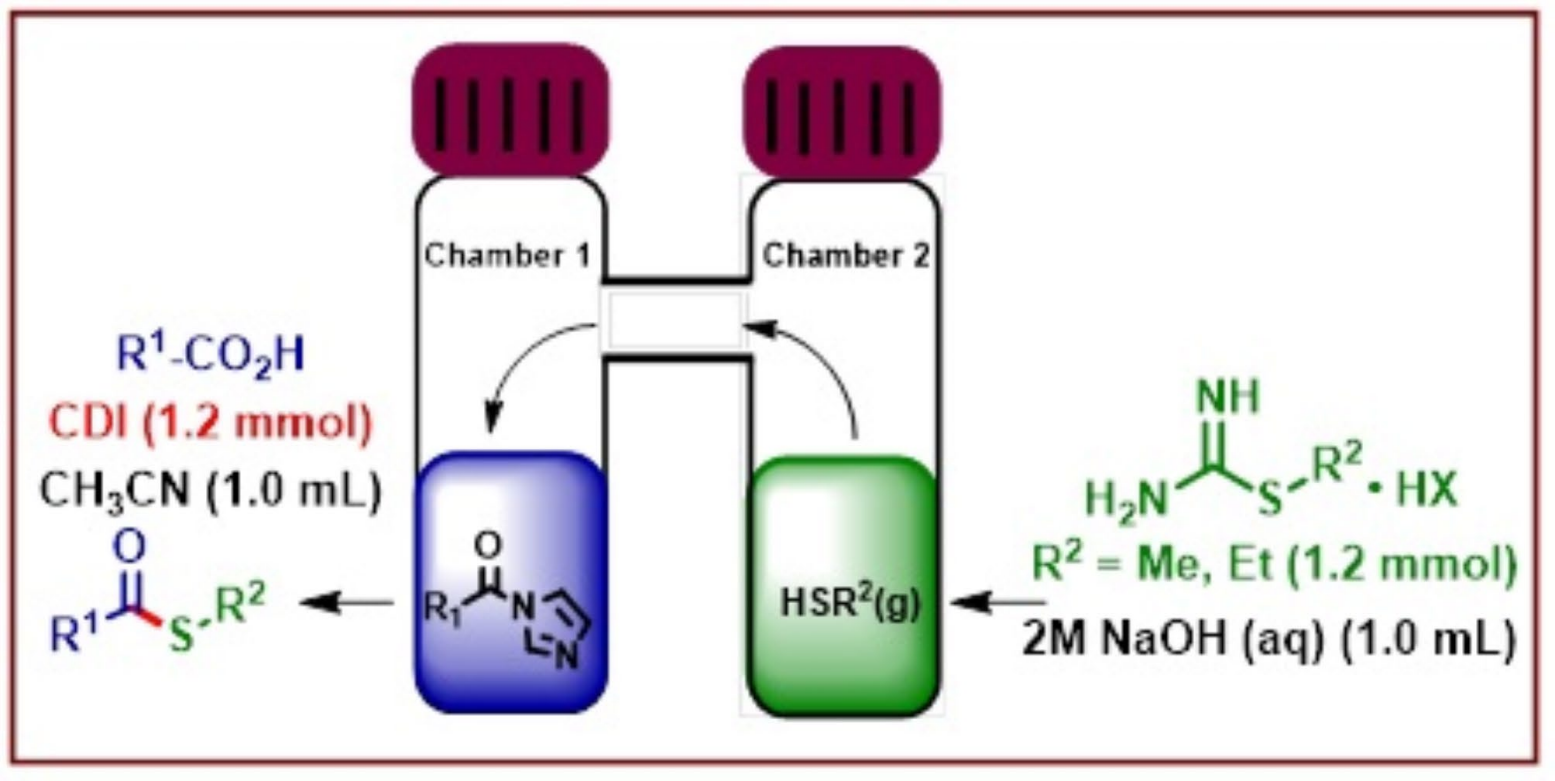
Ex situ gas generation using two-chamber H-tube set-up for *S-*methyl thioester synthesis.

Many reports have emerged in past years detailing the use of *S-*methylisothiourea hemisulfate for thiomethylation of different substrates including a report from our lab wherein heteroarenes were thiomethylated at room temperature although in an in situ synthetic procedure due to the compatibility of the reagents towards the presence of water^30,32,33^. We, therefore decided to combine our previous experiences and develop a simple, ambient temperature *S-*methyl thioester synthesis protocol by using CDI as the coupling reagent and *S-*methylisothiourea hemisulfate as the MeSH surrogate in an ex situ two-chamber (H-tube) procedure (shown above in Fig. 3).

### Reaction optimization

Previously, our group published work on the amidation of carboxylic acids employing an ex situ approach, as well as a study on thio-methylation of chloroheteroarenes via in situ generation of methanethiol gas^30,31^. Building upon these studies, we initiated the optimization of our reaction conditions, leveraging the methodologies established in these prior publications. The screening studies for the conversion of carboxylic acid to *S-*methyl thioester was initiated by reacting benzoic acid (**1a**) with 1.2 eq. of CDI in 1.0 mL of acetonitrile as solvent in chamber 1 at room temperature under a nitrogen atmosphere with the resultant solution stirred for 1 h to form the corresponding carbonyl imidazole intermediate of the corresponding carboxylic acid. On completion of an hour with most of the carboxylic acid converted into the intermediate, the second chamber is charged with 0.6 equiv. of thiomethyl-containing reagent, i.e., *S-*methyl thiourea hemisulfate (**2a**) and 1 M 1.0 mL aq. solution of NaOH to generate MeSH in an ex situ fashion. Both the chambers were then sealed to allow the generated MeSH to start reacting with carbonyl imidazole intermediate and the resultant reaction mixture was stirred for 3 h. On work-up of the reaction mass, it was observed that there was no complete conversion of the intermediate formed in chamber 1 to the methyl thioester product and that the product on isolation without using column chromatography furnished only 34% yield (entry 1, Table 1). We initially repeated the experiment using 2.0 mL of 1 M aq. NaOH at the same concentration; however, no significant improvement in yield was observed. (entry 2, Table 1). To further enhance the yield, we increased the concentration of aq. NaOH to 2 M while reducing the volume to 1.0 mL. This adjustment led to the formation of the desired *S-*methyl thioester with a slightly improved yield (entry 3, Table 1). Increasing the amount of 2 M NaOH to 2.0 mL resulted in a further yield increase (entry 4, Table 1). However, the yields remained insufficient for continuation, prompting us to increase the amount of **2a** from 0.6 mmol to 1.2 mmol, along with the addition of 1.0 mL of 2 M NaOH. (entry 5, Table 1). This modification produced a drastic improvement in the yield of the product. Further increase in the amount of aq. NaOH did not affect the yield (entry 6, Table 1). We also increased the amount of CDI to 1.5 mmol under the same conditions, but no significant change in the yield was observed (entry 7, Table 1).

**Table 1:**
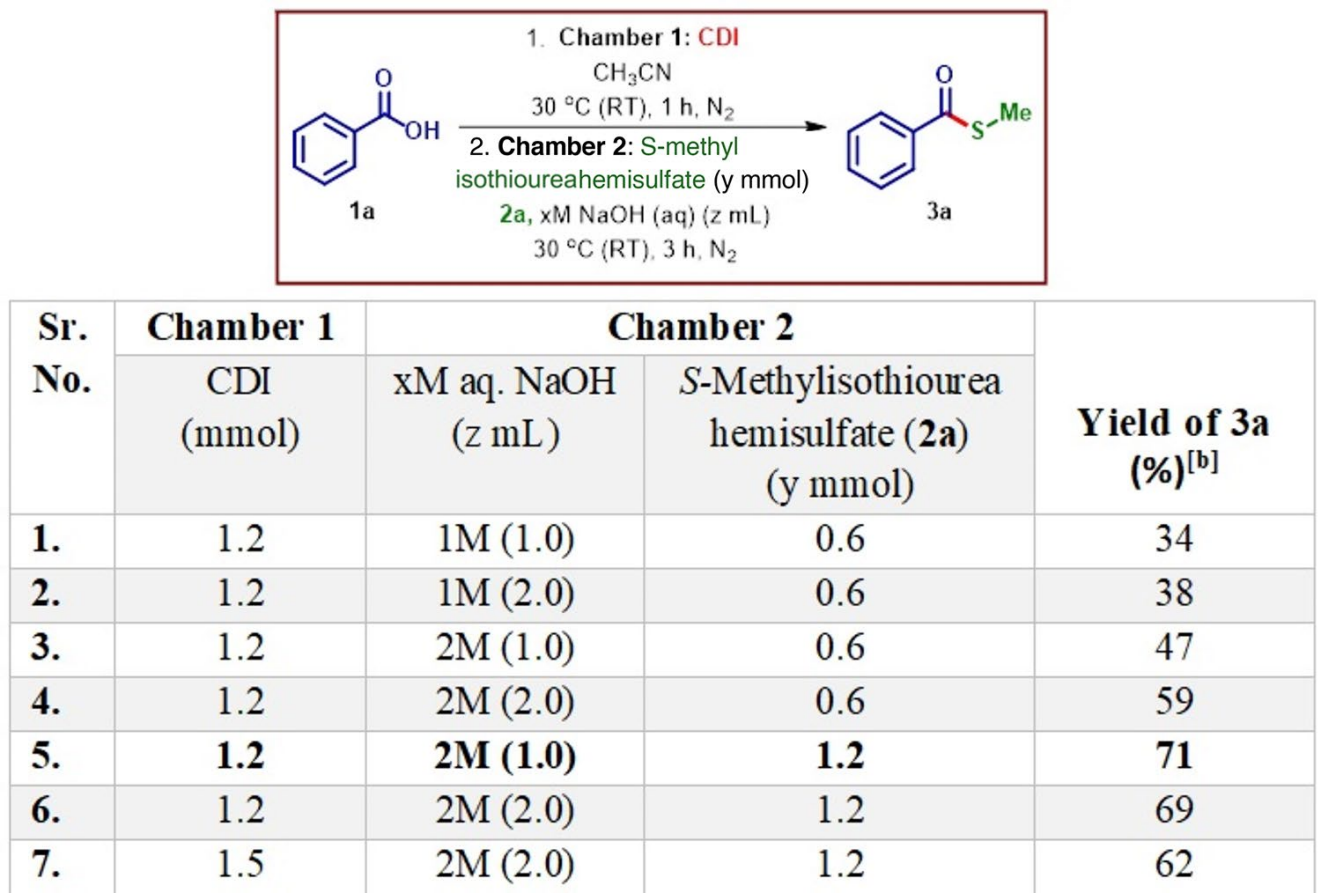
Optimization table.

^[a]^Unless specified, reaction conditions are: In Chamber 1 added 1.0 mmol of carboxylic acid **1a**, 1.2 mmol of CDI, 1.0 mL of CH_3_CN, stirred at 30 °C (RT) for 1 h. After 1 h in Chamber 2, added y mmol of **2a**, z mL of xM NaOH, stirred at 30 °C (RT) for 3 h. ^[b]^Isolated yields.

**Fig. 4:**
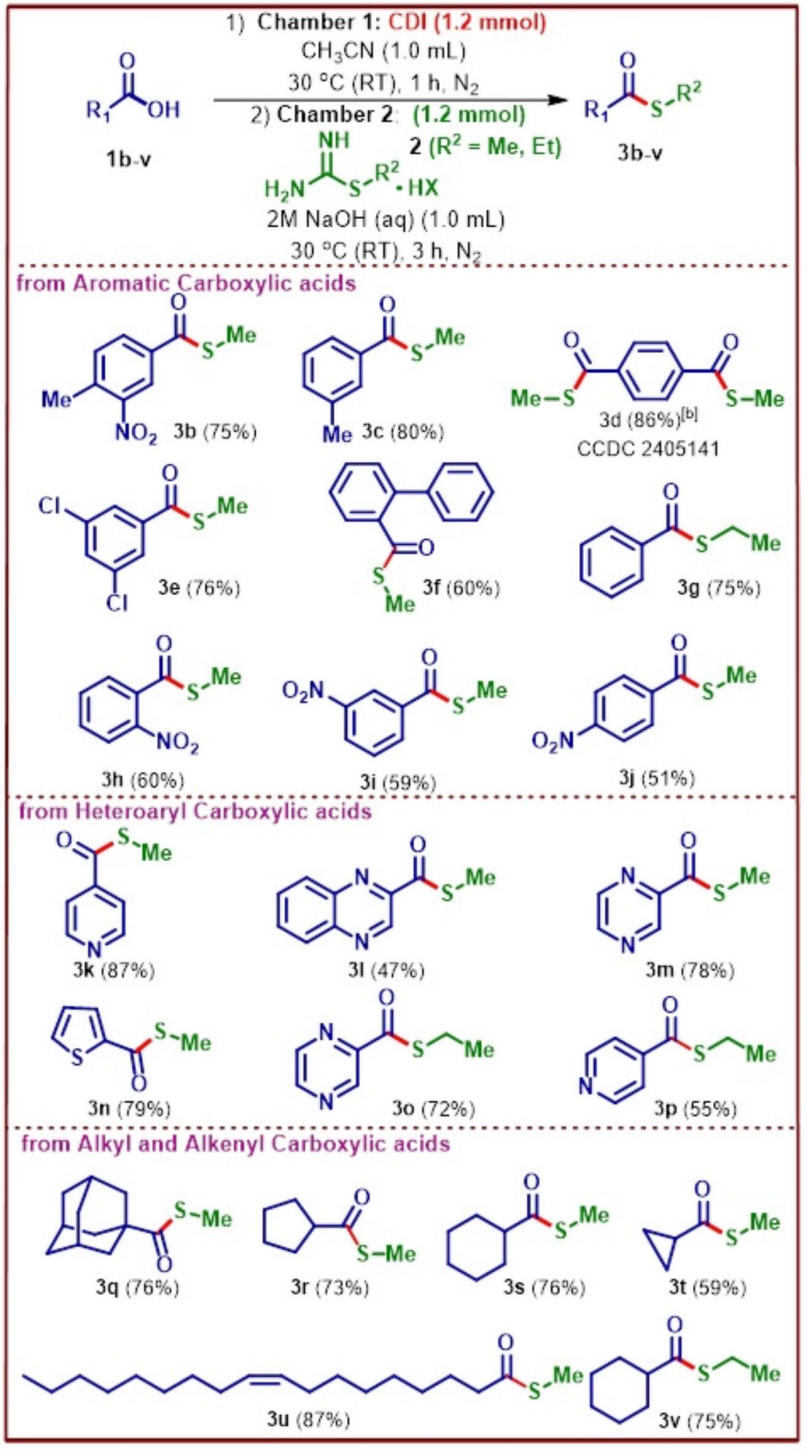
Substrate scope for thiomethylation of carboxylic acids. [a] Unless specified, reaction conditions are: In Chamber 1, was added 1.0 mmol of carboxylic acid, 1.2 mmol of CDI, 1.0 mL of acetonitrile, stirred at 30 °C (RT) for 1 h. After 1 h, in Chamber 2, was added 1.2 mmol of *S-*alkylisothiourea salt, 1.0 mL of 2 M aq. NaOH, stirred at 30 °C (RT) for 3 h. [b] In Chamber 1, was added 2.4 mmol of CDI and in Chamber 2, was added 2.4 equiv. of *S-*alkylisothiourea salt, 2.0 mL of 2 M aq. NaOH.

The optimized *S-*methyl thioesterification condition was next tested for a wide variety of substrates to check the feasibility of the developed protocol. Differently substituted aromatic carboxylic acids such as 4-methyl-3-nitro benzoic acid, 3-methyl benzoic acid, benzene-1,4-dicarboxylic acid, 3,5-dichloro benzoic acid and 2-phenyl benzoic acid were all subjected to the *S-*methyl thioesterification conditions giving corresponding thioesters **3b–f** good yields (Fig. 4). The same protocol with a slight modification of the thioalkylating reagent, i.e., *S-*ethyl thiourea hemisulfate was used to prepare ethyl thioester of benzoic acid **3g** in good yield. Further, ortho-, meta-, and para-nitrobenzoic acids were subjected to the developed reaction conditions. The ortho and meta derivatives exhibited similar yields of methyl thioesters **3h–i**, while the para-substituted derivative showed a slight decrease in yield of **3j**. This suggests that the position of the substituent has minimal influence on the reactivity of the substrates. Next, we turned our attention to the functionalization of heteroaryl carboxylic acids such as pyridine-4-carboxylic acid, quinoxaline-2-carboxylic acid, pyrazine-2-carboxylic acid, thiophene-2-carboxylic acid to furnish the respective *S-*methyl thioester products **3k–n** in decent to good yields.

**Fig. 5:**
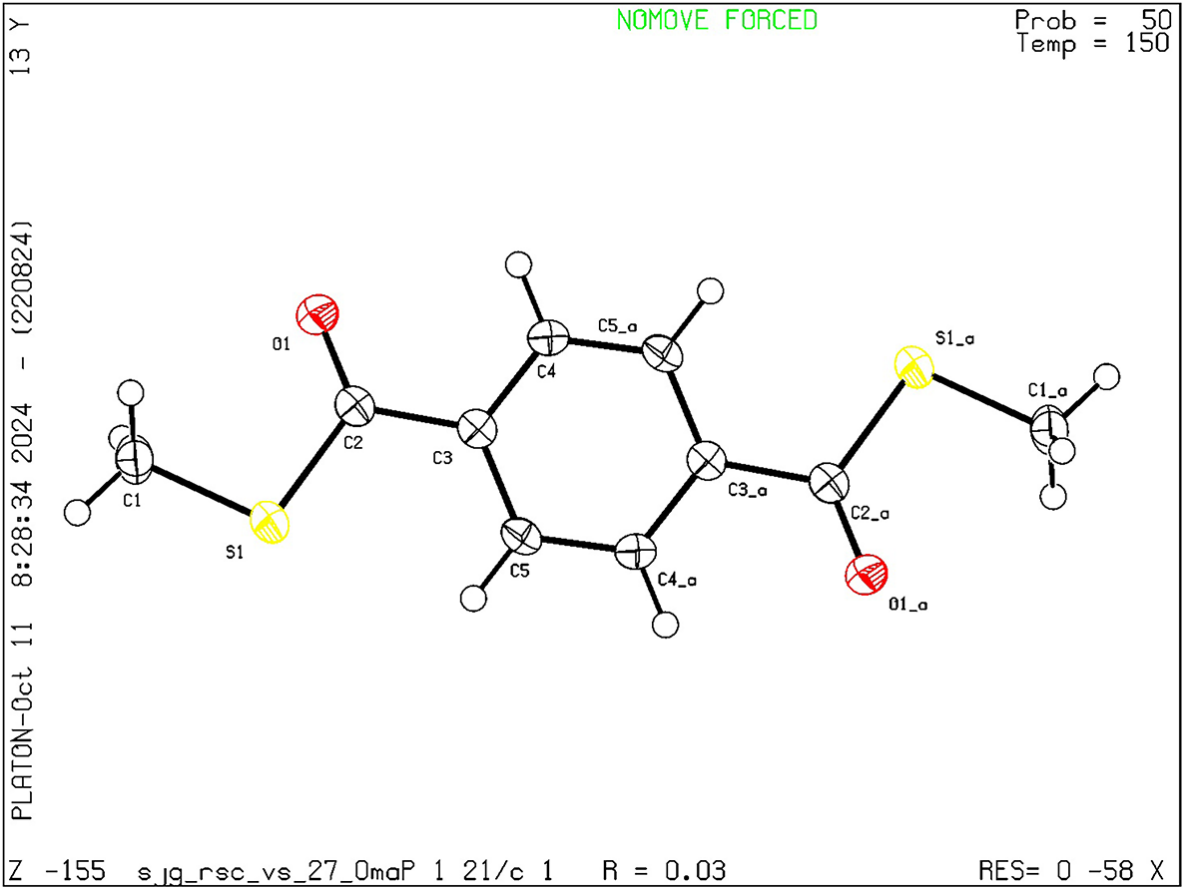
Single Crystal X-ray structure of **3d**. (Ellipsoid is drawn at the 50% Probability level and CCDC- 2405141)

Pyrazine-2-carboxylic acid and pyridine-4-carboxylic acid were further functionalized to their corresponding ethyl thioesters **3o–p** in good yields demonstrating the applicability of the developed protocol. Alkyl carboxylic acids, especially 1-adamantanecarboxylic acid, 1-cyclopentyl carboxylic acid and 1-cyclohexyl carboxylic acid reacted effectively with the thiomethylating reagent in the presence of CDI in an ex situ fashion to provide good yields of the desired *S-*methyl thioester products **3q–s**. Synthetically more challenging substrate, 1-cyclopropyl carboxylic acid that can readily undergo ring opening due to the large angle strain present on the 3-membered ring (Baeyer’s strain) when subjected to the reaction conditions yielded decent amount of the product **3t**. Finally, alkenyl carboxylic acid such as oleic acid was employed as the substrate wherein excellent yield of the desired product **3u** was obtained. Also, *S-*ethyl derivative of cyclohexane carboxylic acid **3v** was synthesised with good yield. The *S-*methyl thioester derivative of terephthalic acid exhibited a crystalline structure, enabling the formation of single crystals ideal for X-ray analysis. X-ray structure details for the *S-*methyl thioester derivatives of terephthalic acid as shown below in Fig. 5.

**Fig. 6:**
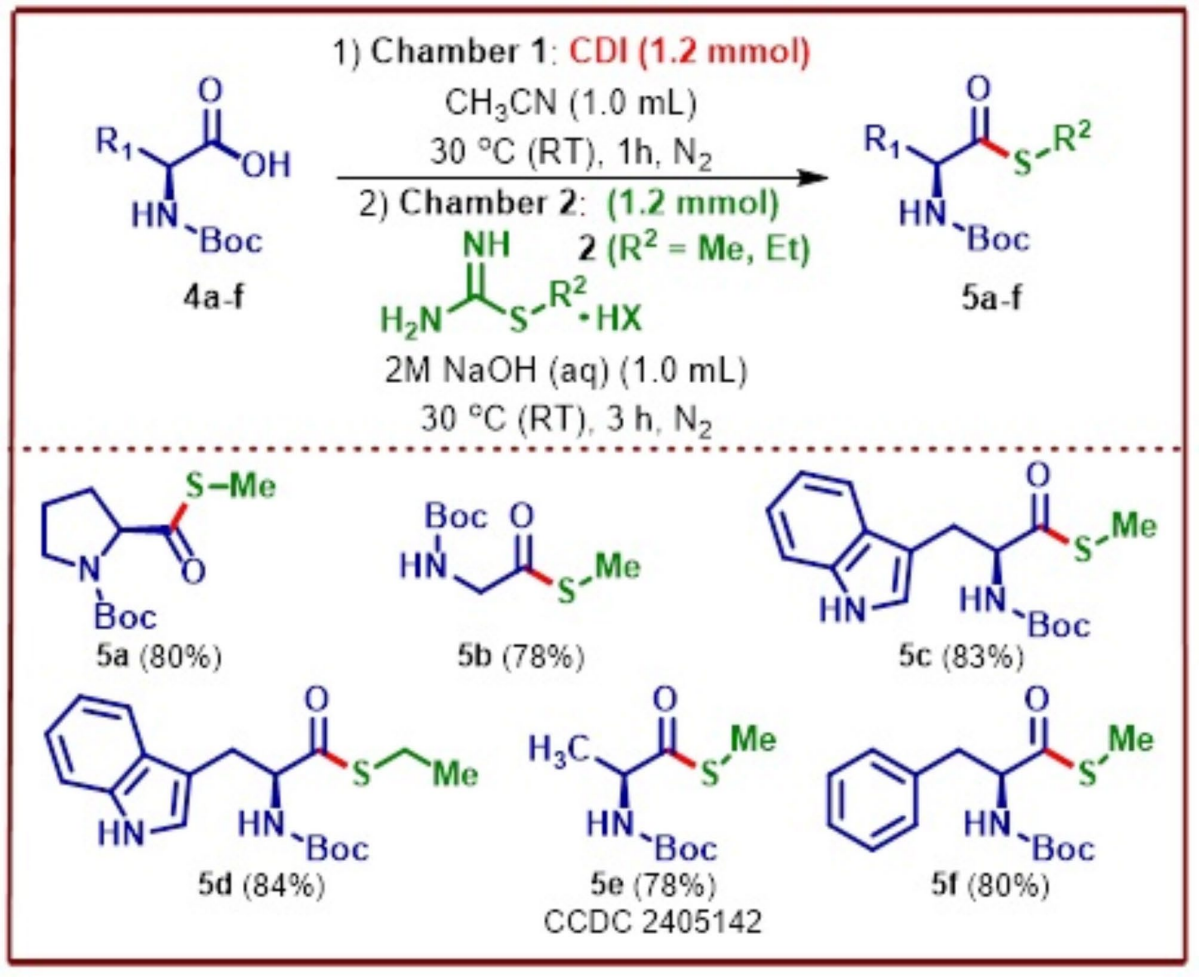
Synthesis of *S-*methyl thioesters of α-amino acids. [a] In Chamber 1 added 1.0 mmol of amino acid, 1.2 mmol of CDI, 1.0 mL of CH_3_CN, stirred at 30 °C (RT) for 1 h. After 1 h, in chamber 2 added 1.2 mmol of *S-*alkylisothiourea salt, 1.0 mL of 2 M NaOH, stirred at 30 °C (RT) for 3 h.

**Fig. 7:**
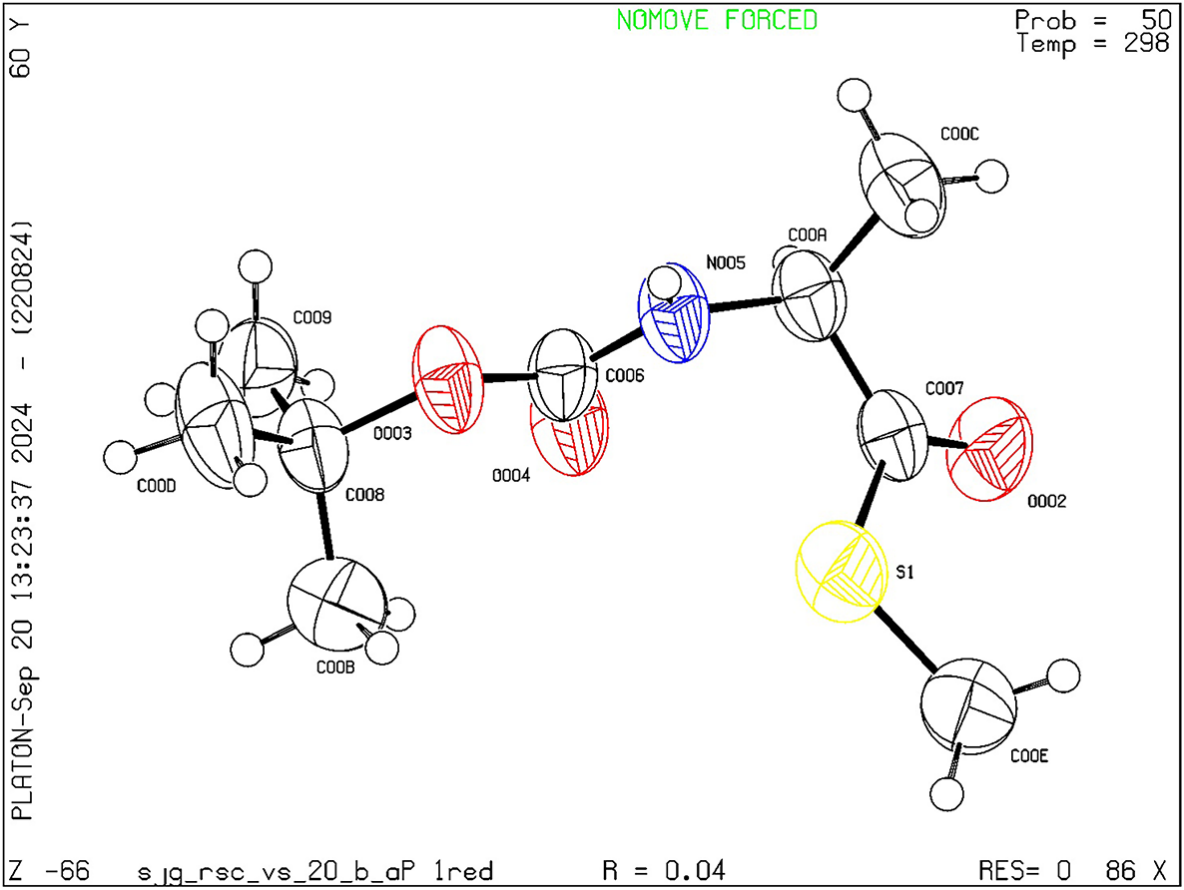
Single crystal X-ray structure of **5e**. (Ellipsoid is drawn at the 50% Probability level and CCDC- 2405142)

α-Amino acid derivatization is an important synthetic strategy leading to the development of unnatural amino acids that has in recent years revolutionalized the area of protein engineering due to the enhanced stability offered by these molecules^34,35^. Thioester derivatives of α-amino acids on the other hand can act as useful synthetic intermediates for peptide synthesis and have been synthesized previously by Bonnarme and co-workers^36^ although using brevibacteria *via* an enzymatic pathway rather than synthetic. Given the shortcomings related to the above-mentioned synthetic methodologies for obtaining *S-*methyl thioesters of α-amino acids, we decided to explore the possibility of obtaining these important synthetic intermediates using our developed protocol. Accordingly, *N*-Boc protected L-proline, glycine, L-tryptophan, L-alanine and L-phenylalanine were subjected to the developed ex situ S-methyl thioesterification conditions providing in all the cases good to excellent yields of the desired products **5a–f** (Fig. 6). Further, the polarimeter data revealed no changes in the absolute configuration of α-amino acids, confirming that there is a retention of configuration. The structure of *S-*methyl thioester derivative of *N*-Boc protected alanine **5e** was unambiguously confirmed by single crystal XRDS (Fig. 7).

**Fig. 8:**
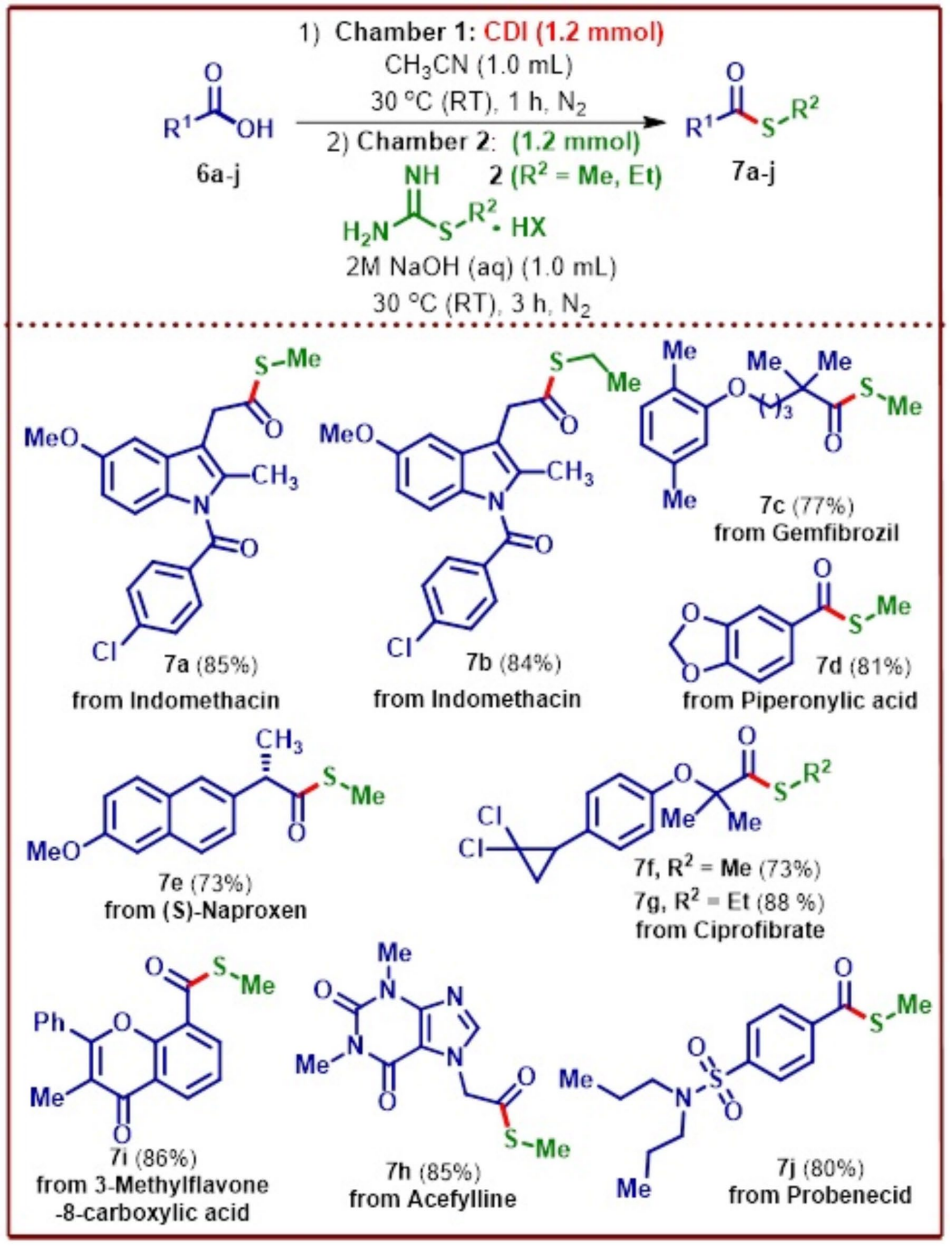
Late-stage *S-*methyl thioesterification of bioactive natural products and pharmaceutical drugs. [a] In Chamber 1, was added 1.0 mmol of carboxylic acid, 1.2 mmol of CDI, 1.0 mL of CH_3_CN, stirred at 30 °C (RT) for 1 h. After 1 h, in Chamber 2, was added 1.2 mmol of *S-*alkylisothiourea salt, 1.0 mL of 2 M aq. NaOH, stirred at 30 °C (RT) for 3 h.

Late-stage functionalization strategies have been employed more frequently in recent years by medicinal chemists as a powerful tool in drug discovery readily providing access to molecules with improved properties. For a synthetic methodology to be able to demonstrate a late-stage functionalization strategy can certainly find utility in drug discovery programs. Encouraged by the results obtained with the *S-*methyl thioesterification of α-amino acids, a late-stage strategy to functionalize bio-active compounds such as pharmaceutical drugs, natural products and agrochemicals was next undertaken to demonstrate the capability of the developed methodology to go beyond the simple synthetic substrates given the mild reaction conditions. Indomethacin^37^, an anti-inflammatory drug used commonly for the treatment of arthritis, and tendonitis when subjected to the thioesterification conditions to incorporate *S-*methyl and *S-*ethyl functionality, gave excellent yields of the respective thioester products **7a–b** (Fig. 8). Gemfibrozil^38^, a drug that has been administered to reduce cholesterol and triglycerides in the blood leading to the reduction in the possibility of heart related issues was next to undergo ex situ S-methyl thioesterification to furnish the functionalized product **7c** in 77% yield.

Piperonylic acid is a naturally occurring bioactive molecule isolated from several plants and has been studied extensively for its epidermal growth factor properties that could make it an important component of cosmetics or ointments prescribed for skin wound healing^39^. Subjecting piperonylic acid to the ex situ S-methyl thioester conditions provided 81% of the desired product **7d**. *S-*Naproxen was the next pharmaceutical drug with promising anti-inflammatory properties and has been designated as a non-steroidal drug^40^. It has been routinely used for the treatment of menstrual cramps, fever, and rheumatoid arthritis. Being a chiral drug, performing *S-*methyl thioesterification is a challenging proposition but the mildness of the developed product furnished the desired product **7e** in 73% with complete retention in configuration. Ciprofibrate is an important fibric acid derivative commonly administered as a drug for the reduction of triglycerides while increasing high-density lipoprotein (HDL) cholesterol in a condition called as hyperlipidemias^41^. This drug also underwent facile *S-*methyl and *S-*ethyl thioesterification giving the corresponding products **7f–g** in good to excellent yields. Acefylline^42^, an efficient bronchodilator was next treated under the ex situ conditions to give 85% of the *S-*methyl thioester derivative **7h**. Finally, 3-methylflavone-8-carboxylic acid and probenecid were also derivatized effectively to the corresponding *S-*methyl thioester products **7i–j** in good to excellent yields demonstrating the usefulness of the protocol that can tolerate a wide variety of functionalities.

**Fig. 9:**
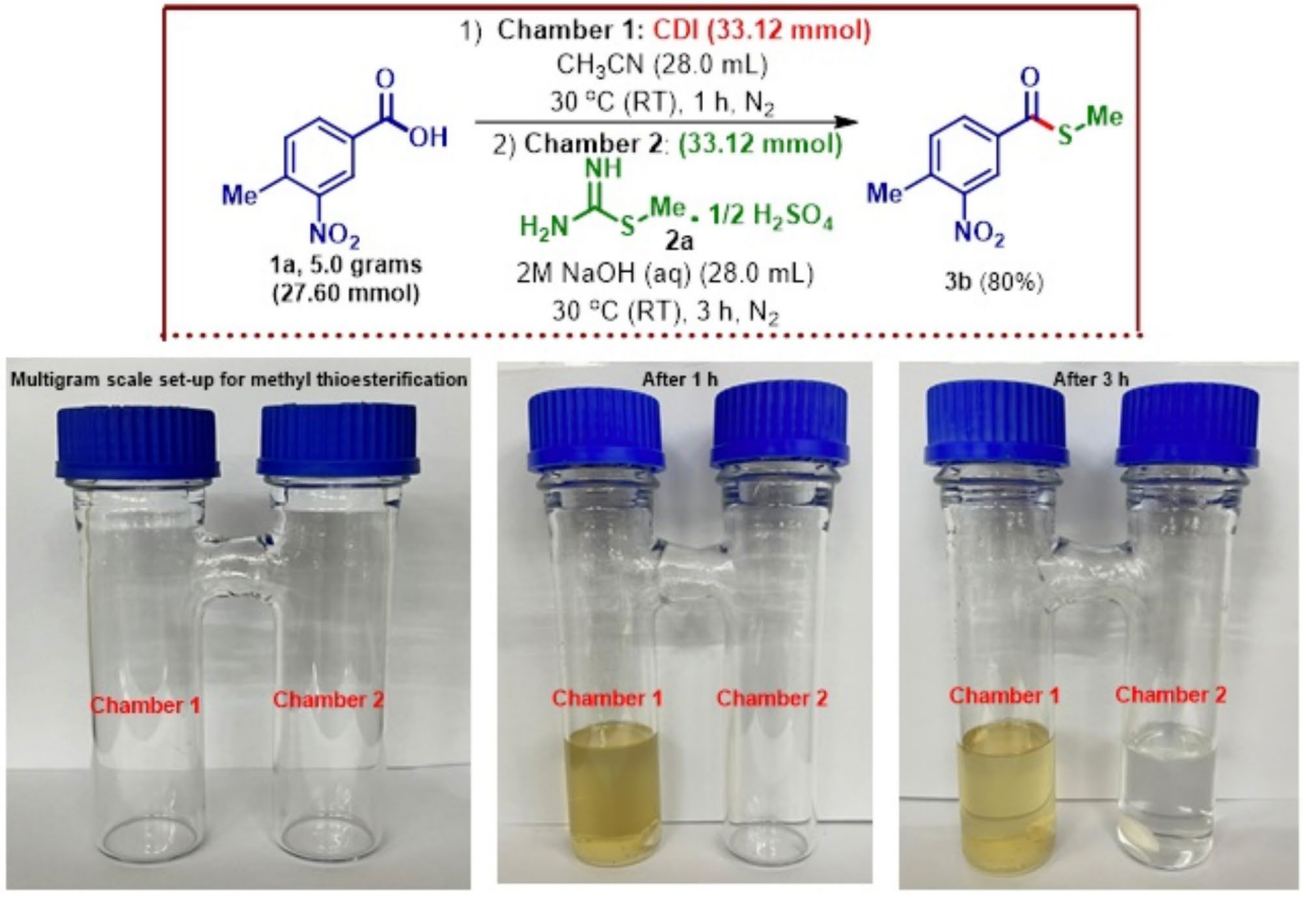
5.0 g-scale of methyl thioesterification of 4-methyl-3-nitro benzoic acid.

All these results certainly point towards the effectiveness of the methyl thioesterification protocol. However, there has always been a question regarding the scalability and reproducibility of the ex situ synthetic transformation given the limitation of the set-up. To address this issue, a multigram-scale (200 mL capacity) set-up was constructed, and a 5-grams scale-up synthesis of methyl thioester of 4-methyl-3-nitro benzoic acid was carried out using the described procedure (FIGURE 9). Gratifyingly, 4.7 g of the methyl thioester product **3b** corresponding to 80% yield was obtained demonstrating the scalability of the protocol, which is very much essential for further applications to the functionalization of small volume high cost synthetic and pharmaceutical intermediates.

## Conclusion

*S-*Methyl thioesters are useful synthetic intermediates and their further conversion to important molecules via simple synthetic transformations makes them ideal candidates for exploration of efficient protocols for their preparation. Metal-mediated as well as metal-free protocols have been found to exhibit limitations pertaining to the cost of the involved reagents, limited substrate scope and poor yields. To address this issue, we have reported herein an ambient temperature protocol involving the ex situ generation of MeSH that rapidly reacts with the active ester formed by the reaction of a variety of carboxylic acids with 1,1-carbonyldiimidazole. A broad substrate scope ranging from aromatic carboxylic acids, heteroaryl carboxylic acids to alkyl or cycloalkyl carboxylic acids was achieved with good to excellent yields obtained in most cases. The mild nature of the developed reactions conditions is ideal in promoting the *S-*methyl thioester synthesis of several chiral amino acids with complete retention in configuration. Late-stage functionalization of bioactive natural products and pharmaceutical drugs was also achieved in good yields while a scale-up protocol involving the conversion of 5.0 g of 4-methyl-3-nitro benzoic acid to its *S-*methyl ester was also successfully performed portraying the scalability of the ex situ protocol.

## Methods

### General procedure A for the synthesis of 3a-3v, 5a-5f and 7a-7j

A clean and dry H-tube with a magnetic stirrer bar was evacuated under vacuum followed by nitrogen flushing three times. In chamber 1, 1.0 mmol of carboxylic acid derivatives, 1.2 mmol of 1,1’-carbonyldiimidazole were added and dissolved in 1.0 mL acetonitrile under nitrogen atmosphere. The reaction was allowed to stir at room temperature for 1 h. After 1 h, in chamber 2 was added 1.2 mmol of *S-*methylisothiourea hemisulfate salt or *S-*ethylisothiourea hydrobromide, followed by addition of 1.0 mL of 2 M aq. NaOH solution under nitrogen atmosphere, and stirred the reaction mixture at 30 ^o^C (Room temperature) for 3 h. (For Compound **3d**, 2.4 mmol of 1,1’-carbonyldiimidazole and 2.4 mmol of *S-*Methylisothiourea hemisulfate in chamber 1, and 2.0 mL of 2 M aq. NaOH solution were added in chamber 2). The progress of the reaction was monitored by TLC. After the completion of the reaction, the reaction mixture was concentrated, quenched with NaHCO_3_ and extracted with CH_2_Cl_2_ (3 × 10 mL). The extract was dried over anhydrous Na_2_SO_4_ and evaporated on a rotary evaporator under a low vacuum, and the desired products were obtained. Compounds (**3f**, **3n**, **3u**, **7e**, **7h**) were further purified using column chromatography.

### General procedure B for scale-up synthesis of 3b

A clean and dry H-tube with a magnetic stirrer bar was evacuated under vacuum followed by nitrogen flushing three times. In chamber 1, 27.60 mmol of carboxylic acid derivatives, 33.12 mmol of 1,1’-carbonyldiimidazole were added and dissolved in 28.0 mL acetonitrile under nitrogen atmosphere. The reaction was allowed to stir at room temperature for 1 h. After 1 h, in chamber 2, was added 33.12 mmol of *S-*Methylisothiourea hemisulfate salt, followed by addition of 28.0 mL of 2 M aq. NaOH solution under nitrogen atmosphere, and the reaction mixture was stirred at 30 °C (Room temperature) for 3 h. The progress of the reaction was monitored by TLC. After completion, the reaction mixture was concentrated, quenched with NaHCO_3_ and extracted with CH_2_Cl_2_. The extract was dried over anhydrous Na_2_SO_4_ and evaporated on a rotary evaporator under a low vacuumto furnish the desired product **3b** (4.7 g, 80%).

## Electronic supplementary material

Below is the link to the electronic supplementary material.

Supplementary Material 1

## Data Availability

“Data is provided within the manuscript or supplementary information files”.
