# Supplementary material for: Room temperature, metal-free, CDI-promoted, ex-situ protocol for S-methyl thioester synthesis
Source: Sci Rep. 2025 May 27;15:18561. doi: 10.1038/s41598-025-97728-3 (PMC12117044; doi:10.1038/s41598-025-97728-3)
Supplement: Supplementary file 1 — Supplementary Material 1 [file 41598_2025_97728_MOESM1_ESM.docx]

**Room Temperature, Metal-Free, CDI-promoted, Ex-situ Protocol for S-Methyl Thioester Synthesis**

Manisha A. Patel,^1^ Vandana Sharma,^1^ Rupali Chavan,^2^ Juhi Pal,^2^ Santosh J. Gharpure,^2^ and

Anant R. Kapdi^1,*^

^1^Department of Chemistry, Institute of Chemical Technology, Nathalal Parekh road, Matunga, Mumbai-400019, India.

^2^Department of Chemistry, Indian Institute of Technology Bombay, Powai, Mumbai-400076, India

**Inhalt**

1. General information……………………………………………………………………...S2

2. Plausible reaction mechanism……………………………………………………………S2

3. Analytical data of substrates…………………………………………………………...…S3-S12

3.1 Thiomethylation of carboxylic acids...…………………………………………S3-S7

3.2 Thiomethylation of aminoacids………………………………………………...S7-S9

3.3 Thiomethylation of drug molecules ...…………………………………………S9-S13

4. References………………………………………………………………………………...S14

5. ^1^H and ^13^C Spectral Data……………………………………………………………….....S15-S52

6. X-ray Crystal data………………………………………………………………...…S53-S55

7. Controlled experiment NMR spectras……………………………………………………S56-S63

**1. General Information:**

All the reactions were carried out under nitrogen atmosphere using oven dried schlenk tube. 2-Methyl-2-thiopseudourea hemisulfate was purchased from zeta scientific. All other reagents were purchased and were used without further purification unless otherwise stated. ^1^H (500 MHz) and ^13^C (126 MHz) NMR spectra and ^1^H (400 MHz) and ^13^C (101 MHz) NMR spectra were recorded on Bruker Avance 500 spectrometer. Chemical shifts δ are given in ppm and the solvent residual peak (CDCl_3_: ^1^H, δ = 7.27; ^13^C, δ = 77.0 and DMSO-d_6_: ^1^H, δ = 2.50; ^13^C, δ = 40) were used as an internal standard. Peak multiplicities are specified as followed: s, singlet; d, doublet; t, triplet; q, quartet; m, multiplet; br, broad. Reactions were monitored by thin layer chromatography (TLC) on Merck silica gel 60 F254 precoated plates and visualized under UV light. anhydrous sodium sulphate (Na_2_SO_4_) was used to dry organic extracts. Solvents were removed using a rotary evaporator under pressure. Crude products obtained were purified by column chromatography on silica gel 60-120 mesh using a mixture of pet ether and ethyl acetate or dichloromethane and methanol. High-resolution mass spectrometry (HRMS) was performed on a Bruker Maxis Impact QTOF MS with ESI source. X-ray diffraction studies were carried out using Bruker Single Crystal Kappa Apex II. For crystal growth, compound was added to a 5 mL glass vial and dissolved in dichloromethane:chloroform (1:1). Then, the glass vial was placed in a dry, ventilated place at room temperature for few days. Optical rotations were measured on Rudolph Research Analytical Autopol IV automatic polarimeter.

**2. Plausible reaction mechanism:**

**Figure 1:** Plausible mechanism for CDI-mediated thioesterification of carboxylic acids.^1–4^

**3. Analytical data of substrates**

**3.1 Thiomethylation of Carboxylic acids:**

***S*-methyl benzothioate (3a)**

Product **3a** was prepared according to the general procedure A (108 mg, 71% yield), and obtained as a pale-yellow liquid.

^1^H NMR (500 MHz, CDCl_3_) δ 7.98-7.95 (m, 2H), 7.58-7.54 (m, 1H), 7.46-7.42 (m, 2H), 2.47 (s, 3H).

^13^C{^1^H} NMR (126 MHz, CDCl_3_) δ 192.4, 137.0, 133.2, 128.5, 127.1, 11.7.

HRMS: ESI, [M+H]^+^ Calcd. For (C_8_H_9_OS): 153.0369. Found: 153.0367. The compound exhibited identical ^1^H and ^13^C {^1^H} NMR data to previous reports.^5–7^

**S-methyl 4-methyl-3-nitrobenzothioate (3b)**

Product **3b** was prepared according to the general procedure A (158 mg, 75% yield), and obtained as a light brown solid.

Melting Point = 65-67 ^o^C.

^1^H NMR (500 MHz, CDCl_3_) δ 8.55 (s, 1H), 8.07 (d, *J* = 8.0 Hz, 1H), 7.46 (d, *J* = 8.0 Hz, 1H), 2.67 (s, 3H), 2.52 (s, 3H).

^13^C {^1^H} NMR (126 MHz, CDCl_3_) δ 190.1, 149.2, 138.6, 135.9, 133.2, 130.7, 123.3, 20.6, 11.8.

HRMS: ESI, [M+H]^+^ Calcd. For (C_9_H_10_NO_3_S): 212.0376. Found: 212.0382.

***S*-methyl 3-methylbenzothioate (3c)**

Product **3c** was prepared according to the general procedure A (133 mg, 80% yield), and obtained as a pale-yellow liquid.

^1^H NMR (500 MHz, CDCl_3_) δ 7.77 (s, 2H), 7.38-7.31 (m, 2H), 2.46 (s, 3H), 2.40 (s, 3H).

^13^C {^1^H} NMR (126 MHz, CDCl_3_) δ 192.5, 138.4, 137.0, 134.0, 128.4, 127.5, 124.3, 21.2, 11.7.

HRMS: ESI, [M+H]^+^ Calcd. For (C_9_H_11_OS):167.0525. Found: 167.0527. The compound exhibited identical ^1^H and ^13^C {^1^H} NMR data to previous reports. ^5^

***S,S*-dimethyl benzene-1,4-bis(carbothioate) (3d)**

Product **3d** was prepared according to the general procedure A (195 mg, 86 % yield), and obtained as a white crystalline solid.

Melting Point = 112-114 ^o^C.

^1^H NMR (500 MHz, CDCl_3_) δ 8.03 (s, 2H), 2.51 (s, 3H).

^13^C {^1^H} NMR (126 MHz, CDCl_3_) δ 191.7, 140.4, 127.3, 11.9.

HRMS: ESI, [M+H]^+^ Calcd. For (C_10_H_11_O_2_S_2_): 227.0195. Found: 227.0198. The compound exhibited identical ^1^H and ^13^C {^1^H} NMR data to previous reports.^7^

***S-*ethyl 3,5-dichlorobenzothioate (3e)**

Product **3e** was prepared according to the general procedure A (168 mg, 76 % yield), and obtained as a dark-yellow liquid.

^1^H NMR (500 MHz, CDCl_3_) δ 7.60 (d, *J* = 8.3 Hz, 1H), 7.46 (d, *J* = 1.9 Hz, 1H), 7.30 (dd, *J* = 8.3, 2.0 Hz, 1H), 2.49 (s, 3H).

^13^C {^1^H} NMR (126 MHz, CDCl_3_) δ 203.1, 52.6, 29.5, 25.6, 25.5, 22.8, 14.7.

HRMS: ESI, [M+H]^+^ Calcd. For (C_8_H_7_Cl_2_OS): 220.9590. Found: 220.9597.

***S*-methyl [1,1'-biphenyl]-2-carbothioate (3f)**

Product **3f** was prepared according to the general procedure A (137 mg, 60 % yield), and obtained as a pale-yellow liquid. Pet ether/ethyl acetate (8.8:1.2) was used as a mobile phase for column chromatography.

^1^H NMR (500 MHz, CDCl_3_) δ 7.71 (d, *J* = 7.4 Hz, 1H), 7.54 (t, *J* = 7.5 Hz, 1H), 7.43-7.35 (m, 7.9 Hz, 7H), 2.35 (s, 3H).

^13^C {^1^H} NMR (126 MHz, CDCl_3_) δ 195.4, 140.3, 140.2, 138.5, 131.1, 130.9, 128.8, 128.2, 128.0, 127.4, 127.1, 12.6.

HRMS: ESI, [M+H]^+^ Calcd. For (C_14_H_13_OS): 229.0682. Found: 229.0684.

***S*-ethyl benzothioate (3g)**

Product **3g** was prepared according to the general procedure A (125 mg, 75% yield), and obtained as a yellow liquid.

^1^H NMR (500 MHz, CDCl_3_) δ 7.96 (d, *J* = 7.6 Hz, 2H), 7.55 (t, *J* = 7.4 Hz, 1H), 7.44 (t, *J* = 7.7 Hz, 2H), 3.08 (q, *J* = 7.4 Hz, 2H), 1.35 (t, *J* = 7.4 Hz, 3H).

^13^C {^1^H} NMR (126 MHz, CDCl_3_) δ 192.1, 137.2, 133.2, 128.5, 127.1, 23.4, 14.7.

The compound exhibited identical ^1^H and ^13^C {^1^H} NMR data to previous reports.^8,9^

***S*-methyl 2-nitrobenzothioate (3h)**

Product **3h** was prepared according to the general procedure A (118 mg, 60% yield), and obtained as a pale yellow solid.

^1^H NMR (400 MHz, CDCl_3_) δ 8.00 (t, *J* = 1.6 Hz, 1H), 7.79-7.26 (m, 3H), 2.54 (s, 3H).

^13^C {^1^H} NMR (101 MHz, CDCl_3_) δ 191.8, 134.8, 133.4, 131.8, 128.8,124.8, 12.8.

HRMS: ESI, [M+H]^+^ Calcd. For (C_8_H_8_NO_3_S): 198.0219. Found: 198.0208.

***S*-methyl 3-nitrobenzothioate (3i)**

Product **3i** was prepared according to the general procedure A (116 mg, 59% yield), and obtained as a pale-yellow solid.

^1^H NMR (400 MHz, CDCl_3_) δ 8.79 (d, *J* = 1.2 Hz, 1H), 8.42 (dd, *J* = 7.2 Hz, 1.2 Hz, 1H), 8.28 (t, *J* = 0.8 Hz, 1H), 7.67 (t, *J* = 8.0 Hz, 1H), 2.55 (s, 3H).^13^C {^1^H} NMR (101 MHz, CDCl_3_) δ 190.5, 138.5, 132.8, 130.0, 127.6, 122.3, 12.2.

The compound exhibited identical ^1^H and ^13^C {^1^H} NMR data to previous reports.^5^

***S*-methyl 4-nitrobenzothioate (3j)**

Product **3j** was prepared according to the general procedure A (100 mg, 51% yield), and obtained as a pale-yellow Solid.

^1^H NMR (400 MHz, CDCl_3_) δ 8.30 (d, *J* = 8.8 Hz, 2H), 8.11 (d, *J* = 9.2 Hz, 2H) 2.54 (s, 3H).

^13^C {^1^H} NMR (101 MHz, CDCl_3_) δ 191.0, 150.6, 141.7, 128.3, 124.0,12.3.

The compound exhibited identical ^1^H and ^13^C {^1^H} NMR data to previous reports.^10^

***S*-methyl pyridine-4-carbothioate (3k)**

Product **3k** was prepared according to the general procedure A (133 mg, 87% yield), and obtained as a white crystalline solid.

^1^H NMR (500 MHz, CDCl_3_) δ 8.79 (d, *J* = 5.8 Hz, 2H), 7.76 (d, *J* = 5.8 Hz, 2H), 2.52 (s, 3H).

^13^C {^1^H} NMR (126 MHz, CDCl_3_) δ 191.6, 150.7, 143.0, 120.2, 11.7

The compound exhibited identical ^1^H and ^13^C {^1^H} NMR data to previous reports.^11^

***S*-methyl quinoxaline-2-carbothioate (3l)**

Product **3l** was prepared according to the general procedure A (96 mg, 47% yield), and obtained as a brown solid.

^1^H NMR (500 MHz, CDCl_3_) δ 9.46 (s, 1H), 8.27 (d, *J* = 8.2 Hz, 1H), 8.21 (d, *J* = 8.1 Hz, 1H), 7.95-7.87 (m, 2H), 2.55 (s, 3H).

^13^C {^1^H} NMR (126 MHz, CDCl_3_) δ 192.4, 144.3, 143.6, 140.5, 140.0, 131.3, 130.0, 129.3, 128.4, 10.5.

HRMS: ESI, [M+H]^+^ Calcd. For (C_10_H_5_N_2_OS): 205.0430. Found: 205.0435.

***S*-methyl pyrazine-2-carbothioate (3m)**

Product **3m** was prepared according to the general procedure A (120 mg, 78% yield), and obtained as a pale-yellow solid.

^1^H NMR (500 MHz, CDCl_3_) δ 9.21-9.16 (m, 1H), 8.82 (d, *J* = 2.2 Hz, 1H), 8.67 (s, 1H), 2.49 (s, 3H).

^13^C {^1^H} NMR (126 MHz, CDCl_3_) δ 192.9, 148.6, 146.4, 143.7, 141.7, 11.5.

The compound exhibited identical ^1^H and ^13^C {^1^H} NMR data to previous reports.^11^

***S*-methyl thiophene-2-carbothioate (3n)**

Product **3n** was prepared according to the general procedure A (125 mg, 79% yield), and obtained as a yellow liquid. Pet ether / ethyl acetate (9:1) was used as a mobile phase for column chromatography.

^1^H NMR (500 MHz, CDCl_3_) δ 7.79 (d, *J* = 3.5 Hz, 1H), 7.60 (d, *J* = 4.8 Hz, 1H), 7.10 (t, *J* = 4.3 Hz, 1H), 2.48 (s, 3H).

^13^C {^1^H} NMR (126 MHz, CDCl_3_) δ 184.4, 142.0, 132.4, 130.8, 127.8, 11.7.

HRMS: ESI, [M+H]^+^ Calcd. For (C_6_H_7_OS_2_):158.9933. Found:158.9934. The compound exhibited identical ^1^H and ^13^C {^1^H} NMR data to previous reports. ^5,6^

***S*-ethyl pyrazine-2-carbothioate (3o)**

Product **3o** was prepared according to the general procedure A (121 mg, 72% yield), and obtained as a pale-yellow liquid.

^1^H NMR (500 MHz, CDCl_3_) δ 9.16 (d, *J* = 1.2 Hz, 1H), 8.79 (d, *J* = 2.4 Hz, 1H), 8.64 (dd, *J* = 2.3, 1.5 Hz, 1H), 3.07 (q, *J* = 7.4 Hz, 2H), 1.36 (t, *J* = 7.4 Hz, 3H).

^13^C {^1^H} NMR (126 MHz, CDCl_3_) δ 192.5, 148.6, 146.6, 143.7, 141.8, 23.1, 14.4.

HRMS: ESI, [M+H]^+^ Calcd. For (C_7_H_9_N_2_OS): 169.04301. Found: 169.0432.

***S*-ethyl pyridine-4-carbothioate (3p)**

Product **3p** was prepared according to the general procedure A (92 mg, 55% yield), and obtained as a brown liquid.

^1^H NMR (500 MHz, CDCl_3_) δ 8.74 (d, J = 1.6 Hz, 1H), 8.73 (d, J = 1.6 Hz, 1H), 7.71 (d, J = 1.6 Hz, 1H), 7.70 (d, J = 1.6 Hz, 1H), 3.07 (q, J = 7.4 Hz, 2H), 1.33 (t, J = 7.4 Hz, 3H).

^13^C {^1^H} NMR (126 MHz, CDCl_3_) δ 191.3, 150.7, 143.2, 120.2, 23.7, 14.5.

***S*-methyl (*3r*,*5r*,*7r*)-adamantane-1-carbothioate (3q)**

Product **3q** was prepared according to the general procedure A (160 mg, 76% yield), and obtained as a pale-yellow liquid.

^1^H NMR (500 MHz, CDCl_3_) δ 2.16 (s, 3H), 1.98 (s, 3H), 1.85 (d, *J* = 2.3 Hz, 6H), 1.65 (q, *J* = 12.4 Hz, 6H).

^13^C {^1^H} NMR (126 MHz, CDCl_3_) δ 207.0, 48.3, 39.2, 36.4, 28.1, 10.9.

HRMS: ESI, [M+H]^+^ Calcd. For (C_12_H_19_OS): 211.1151. Found: 211.1157. The compound exhibited identical ^1^H and ^13^C {^1^H} NMR data to previous reports.^5^

***S*-methyl cyclopentanecarbothioate (3r)**

Product **3r** was prepared according to the general procedure A (105 mg, 73% yield), and obtained as a colorless liquid.

^1^H NMR (500 MHz, CDCl_3_) δ 2.99 (p, *J* = 7.9 Hz, 1H), 2.28 (s, 3H), 1.94 – 1.78 (m, 4H), 1.66 (ddd, *J* = 59.2, 15.6, 8.0 Hz, 4H).

^13^C {^1^H} NMR (126 MHz, CDCl_3_) δ 203.4, 53.0, 30.5, 25.8.

The compound exhibited identical ^1^H and ^13^C {^1^H} NMR data to previous reports.^12^

***S-*methyl cyclohexanecarbothioate (3s)**

Product **3s** was prepared according to the general procedure A (120 mg, 76% yield), and obtained as a colorless liquid.

^1^H NMR (500 MHz, CDCl_3_) δ 2.42 (tt, *J* = 11.6, 3.5 Hz, 1H), 2.20 (s, 3H), 1.84 (d, *J* = 13.3 Hz, 2H), 1.74 – 1.69 (m, 2H), 1.59 (dd, *J* = 11.1, 3.1 Hz, 1H), 1.44-1.36 (m, 2H), 1.24 – 1.13 (m, 3H).

^13^C {^1^H} NMR (126 MHz, CDCl_3_) δ 202.6, 51.6, 28.5, 24.6, 24.5, 10.2.

The compound exhibited identical ^1^H and ^13^C {^1^H} NMR data to previous reports.^5,11^

***S*-methyl cyclopropanecarbothioate(3t)**

Product **3t** was prepared according to the general procedure A (69 mg, 59% yield), and obtained as a colorless oil.

^1^H NMR (500 MHz, CDCl_3_) δ 2.31 (s, 3H), 2.06-2.01 (m, 1H), 1.18-1.13 (m, 2H), 0.97-0.90 (m, 2H).

^13^C {^1^H} NMR (126 MHz, CDCl_3_) δ 199.7, 22.5, 11.5, 10.6.

***S*-methyl (*Z*)-octadec-9-enethioate (3u)**

Product **3u** was prepared according to the general procedure A (272 mg, 87% yield), and obtained as a pale-yellow oil. Pet ether / ethyl acetate (9.3:0.7) was used as a mobile phase for column chromatography.

^1^H NMR (500 MHz, CDCl_3_) δ 5.39 – 5.32 (m, 2H), 2.57 (t, *J* = 7.5 Hz, 2H), 2.31 (s, 3H), 2.10 – 1.99 (m, 4H), 1.67 (dd, *J* = 14.2, 7.0 Hz, 2H), 1.35 – 1.27 (m, 20H), 0.90 (t, *J* = 6.3 Hz, 3H).

^13^C {^1^H} NMR (126 MHz, CDCl_3_) δ 200.0, 130.0, 129.7, 43.9, 31.9, 29.7, 29.6, 29.5, 29.5, 29.3, 29.1, 29.0, 28.9, 27.2, 27.1, 25.7, 22.7, 14.1, 11.5.

HRMS: ESI, [M+H]^+^ Calcd. For (C_19_H_37_OS): 313.2560. Found: 313.2560.

***S-*ethyl cyclohexanecarbothioate (3v)**

Product **3v** was prepared according to the general procedure A (129 mg, 75% yield), and obtained as a colorless liquid.

^1^H NMR (500 MHz, CDCl_3_) δ 2.87 (q, *J* = 7.4 Hz, 2H), 2.48 (tt, *J* = 11.5, 3.5 Hz, 1H), 1.93 (dd, *J* = 13.5, 2.1 Hz, 2H), 1.83-1.77 (m, 2H), 1.71-1.64 (m, 1H), 1.47 (qd, *J* = 12.3, 3.0 Hz, 2H), 1.33-1.19 (m, 6H).

^13^C {^1^H} NMR (126 MHz, CDCl_3_) δ 203.1, 52.6, 29.5, 25.6, 25.5, 22.8, 14.7.

HRMS: ESI, [M+H]^+^ Calcd. For (C_9_H_17_OS): 173.0995. Found: 173.0999. The compound exhibited identical ^1^H and ^13^C {^1^H} NMR data to previous reports.^12^

**3.2 Thiomethylation of amino acids**

***tert*-butyl (*S*)-2-((methylthio)carbonyl)pyrrolidine-1-carboxylate (5a)**

Product **5a** was prepared according to the general procedure B (196 mg, 80% yield), and obtained as a pale-yellow liquid.

^1^H NMR (500 MHz, CDCl_3_) δ 4.48 (d, *J* = 8.4 Hz, 1H), 4.37 (d, *J* = 8.3 Hz, 1H), 3.60 – 3.51 (m, 2H), 3.51 – 3.38 (m, 2H), 2.28 (s, 3H), 2.27 (s, 3H), 2.24 – 2.01 (m, 4H), 2.00 – 1.88 (m, 4H), 1.49 (s, 9H), 1.42 (s, 9H).

^13^C {^1^H} NMR (126 MHz, CDCl_3_) δ 203.3, 202.8, 154.6, 153.9, 80.4, 80.2, 66.1, 65.8, 46.8, 46.5, 31.5, 30.6, 28.4, 28.2, 24.0, 23.3, 11.4, 11.1.

HRMS: ESI, [M+H]^+^ Calcd. For (C_11_H_20_NO_3_S): 246.1159. Found: 246.1162.

[α]^25^_D_ = – 131.2 (c = 0.5, CHCl_3_).

***S*-methyl 2-((*tert*-butoxycarbonyl)amino)ethanethioate (5b)**

Product **5b** was prepared according to the general procedure B (160 mg, 78% yield), and obtained as a pale-yellow liquid.

^1^H NMR (500 MHz, CDCl_3_) δ 5.19 (s, 1H), 4.05 (d, *J* = 5.9 Hz, 2H), 2.32 (s, 3H), 1.47 (s, 9H).

^13^C {^1^H} NMR (126 MHz, CDCl_3_) δ 198.7, 155.5, 80.3, 50.2, 28.3, 11.16.

HRMS: ESI, [M+H]^+^ Calcd. For (C_8_H_16_NO_3_S): 206.0845. Found: 206.0850. The compound exhibited identical ^1^H and ^13^C {^1^H} NMR data to previous reports.^13^

**S-methyl (*S*)-2-((*tert*-butoxycarbonyl)amino)-3-(1H-indol-3-yl)propanethioate (5c)**

Product **5c** was prepared according to the general procedure B (278 mg, 83% yield), and obtained as a white powder solid.

Melting Point = 148-150 ^o^C.

^1^H NMR (500 MHz, CDCl_3_) δ 8.24 (s, 1H), 7.56 (d, *J* = 7.9 Hz, 1H), 7.35 (d, *J* = 8.1 Hz, 1H), 7.19 (t, *J* = 7.5 Hz, 1H), 7.12 (t, *J* = 7.4 Hz, 1H), 7.01 (s, 1H), 5.04 (d, *J* = 8.3 Hz, 1H), 4.69 (dd, *J* = 13.7, 5.8 Hz, 1H), 3.34 – 3.25 (m*,* 2H), 2.24 (s, 3H), 1.42 (s, 9H).

^13^C {^1^H} NMR (126 MHz, CDCl_3_) δ 202.4, 155.2, 136.1, 127.6, 123.0, 122.2, 119.7, 118.8, 111.2, 109.8, 80.3, 60.6, 28.2, 11.7.

HRMS: ESI, [M+NH_4_]^+^ Calcd. For (C_17_H_26_N_3_O_3_S): 352.1689. Found: 352.1683.

[α]^25^_D_ = – 39.6 (c = 0.5, CHCl_3_).

**S-ethyl (*S*)-2-((*tert*-butoxycarbonyl)amino)-3-(1H-indol-3-yl)propanethioate (5d)**

Product **5d** was prepared according to the general procedure B (293 mg, 84% yield), and obtained as a pale-yellow solid.

Melting Point = 126-128 ^o^C.

^1^H NMR (500 MHz, CDCl_3_) δ 8.35 (s, 1H), 7.57 (d, *J* = 7.9 Hz, 1H), 7.34 (d, *J* = 8.1 Hz, 1H), 7.18 (t, *J* = 7.5 Hz, 1H), 7.11 (t, *J* = 7.4 Hz, 1H), 6.99 (s, 1H), 5.07 (d, *J* = 8.4 Hz, 1H), 4.68 (dd, *J* = 13.7, 5.8 Hz, 1H), 3.34 – 3.23 (m, 2H), 2.83 (dd, *J* = 14.7, 7.3 Hz, 2H), 1.42 (s, 8H), 1.18 (t, *J* = 7.3 Hz, 3H).

^13^C {^1^H} NMR (126 MHz, CDCl_3_) δ 201.9, 155.2, 136.1, 127.6, 123.1, 122.1, 119.6, 118.8, 111.2, 109.7, 80.2, 60.6, 28.3, 28.1, 23.3, 14.3.

HRMS: ESI, [M+H]^+^ Calcd. For (C_18_H_25_N_2_O_3_S): 349.1580. Found: 349.1585.

[α]^25^_D_ = – 32.8 (c = 0.5, CHCl_3_).

**S-methyl (*S*)-2-((*tert*-butoxycarbonyl)amino)propanethioate (5e)**

Product **5e** was prepared according to the general procedure B (171 mg, 78 % yield), and obtained as a pale-yellow solid.

Melting Point = 55-57 ^o^C.

^1^H NMR (500 MHz, CDCl_3_) δ 4.98 (d, *J* = 4.5 Hz, 1H), 4.51 – 4.26 (m, 1H), 2.29 (s, 3H), 1.46 (s, 9H), 1.38 (d, *J* = 7.1 Hz, 3H).

^13^C {^1^H} NMR (126 MHz, CDCl_3_) δ 202.4, 154.9, 80.2, 56.1, 28.3, 18.8, 11.4.

HRMS: ESI, [M+Na]^+^ Calcd. For (C_9_H_17_NNaO_3_S): 242.0821. Found: 242.0825.

[α]^25^_D_ = – 13.6 (c = 0.5, CHCl_3_).

**S-methyl (*S*)-2-((*tert*-butoxycarbonyl)amino)-3-phenylpropanethioate (5f)**

Product **5f** was prepared according to the general procedure B (236 mg, 80% yield), and obtained as a off-white solid.

Melting Point = 92-94 ^o^C.

^1^H NMR (500 MHz, CDCl_3_) δ 7.30 (t, *J* = 7.2 Hz, 2H), 7.24 (d, *J* = 7.4 Hz, 1H), 7.16 (d, *J* = 7.1 Hz, 2H), 4.88 (d, *J* = 8.0 Hz, 1H), 4.64 (dd, *J* = 13.4, 7.4 Hz, 1H), 3.15 (dd, *J* = 14.1, 5.3 Hz, 1H), 3.04 (dd, *J* = 14.0, 7.4 Hz, 1H), 2.28 (s, 3H), 1.41 (s, 9H).

^13^C {^1^H} NMR (126 MHz, CDCl_3_) δ 201.5, 155.0, 135.7, 129.2, 128.6, 127.0, 80.3, 60.9, 38.3, 28.2, 11.6.

HRMS: ESI, [M+H]^+^ Calcd. For (C_15_H_22_NO_3_S): 296.1315. Found: 296.1321.

[α]^25^_D_ = – 20.4 (c = 0.5, CHCl_3_).

**3.3 Thiomethylation of Drug molecules**

***S*-methyl 2-(1-(4-chlorobenzoyl)-5-methoxy-2-methyl-1*H*-indol-3-yl)ethanethioate (7a)**

Product **7a** was prepared according to the general procedure A (330 mg, 85% yield), and obtained as a yellow solid.

Melting Point = 93-95 ^o^C.

^1^H NMR (500 MHz, CDCl_3_) δ 7.67 (d, *J* = 8.4 Hz, 2H), 7.47 (d, *J* = 8.4 Hz, 2H), 6.95 (d, *J* = 2.3 Hz, 1H), 6.88 (d, *J* = 9.0 Hz, 1H), 6.68 (dd, *J* = 9.0, 2.3 Hz, 1H), 3.87 (s, 2H), 3.84 (s, 3H), 2.40 (s, 3H), 2.28 (s, 3H).

^13^C {^1^H} NMR (126 MHz, CDCl_3_) δ 197.6, 168.2, 156.0, 139.3, 136.6, 133.7, 131.2, 130.7, 130.5, 129.1, 114.9, 112.1, 111.8, 101.1, 55.6, 39.1, 13.4, 11.9.

HRMS: ESI, [M+H]^+^ Calcd. For (C_20_H_19_ClNO_3_S): 388.0769. Found: 388.0781.

***S*-ethyl 2-(1-(4-chlorobenzoyl)-5-methoxy-2-methyl-1*H*-indol-3-yl)ethanethioate (7b)**

Product **7b** was prepared according to the general procedure A (338 mg, 84% yield), and obtained as a white solid.

Melting Point = 92-94 ^o^C.

^1^H NMR (500 MHz, CDCl_3_) δ 7.73-7.65 (m, 2H), 7.50 (d, *J* = 8.5 Hz, 2H), 6.97 (d, *J* = 2.4 Hz, 1H), 6.91 (d, *J* = 9.0 Hz, 1H), 6.71 (dd, *J* = 9.0, 2.5 Hz, 1H), 3.87 (s, 2H), 3.86 (s, 3H), 2.89 (q, *J* = 7.4 Hz, 2H), 2.42 (s, 3H), 1.25 (t, *J* = 7.4 Hz, 3H).

^13^C {^1^H} NMR (126 MHz, CDCl_3_) δ 197.3, 168.3, 156.1, 139.3, 136.6, 133.8, 131.2, 130.8, 130.6, 129.1, 115.0, 112.3, 111.8, 101.2, 55.7, 39.4, 23.6, 14.5, 13.5.

HRMS: ESI, [M+H]^+^ Calcd. For (C_21_H_21_ClNO_3_S):402.0925. Found: 402.0928.

***S-*methyl 2,2-dimethyl-5-phenoxypentanethioate (7c)**

Product **7c** was prepared according to the general procedure A (194 mg, 77% yield), and obtained as a colorless liquid.

^1^H NMR (500 MHz, CDCl_3_) δ 7.06 (d, *J* = 7.4 Hz, 1H), 6.71 (d, *J* = 7.4 Hz, 1H), 6.66 (s, 1H), 3.96 (t, *J* = 5.6 Hz, 2H), 2.37 (s, 3H), 2.32 (s, 3H), 2.25 (s, 3H), 1.86 – 1.78 (m, 4H), 1.33 (s, 6H).

^13^C {^1^H} NMR (126 MHz, CDCl_3_) δ 206.8, 156.9, 136.4, 130.3, 123.6, 120.7, 111.9, 67.8, 49.4, 37.5, 25.4, 24.9, 21.4, 15.8, 11.4.

HRMS: ESI, [M+H]^+^ Calcd. For (C_16_H_25_O_2_S): 281.1570. Found: 281.1577.

***S*-methyl benzo[*d*][1,3]dioxole-5-carbothioate (7d)**

Product **7d** was prepared according to the general procedure A (159 mg, 81% yield), and obtained as a orange solid.

^1^H NMR (500 MHz, CDCl_3_) δ 7.60 (dd, *J* = 8.2, 1.5 Hz, 1H), 7.42 (d, *J* = 1.4 Hz, 1H), 6.84 (d, *J* = 8.2 Hz, 1H), 6.05 (s, 2H), 2.45 (s, 3H).

^13^C {^1^H} NMR (126 MHz, CDCl_3_) δ 190.7, 151.8, 147.9, 131.5, 123.1, 107.9, 107.1, 101.9, 11.7.

HRMS: ESI, [M+H]^+^ Calcd. For (C_9_H_9_O_3_S): 197.0267. Found: 197.0272. The compound exhibited identical ^1^H and ^13^C {^1^H} NMR data to previous reports.^5^

***S*-methyl 2-(6-methoxynaphthalen-2-yl)propanethioate (7e)**

Product **7e** was prepared according to the general procedure A (190 mg, 73% yield), and obtained as a white solid. Pet ether / ethyl acetate (9:1) was used as a mobile phase for column chromatography.

^1^H NMR (500 MHz, CDCl_3_) δ 7.74-7.68 (m, 3H), 7.40 (dd, *J* = 8.5, 1.4 Hz, 1H), 7.17-7.09 (m, 2H), 4.03 (q, *J* = 7.0 Hz, 1H), 3.91 (s, 3H), 2.25 (s, 3H), 1.62 (s, 3H).

^13^C {^1^H} NMR (126 MHz, CDCl_3_) δ 201.7, 157.7, 134.9, 133.8, 129.3, 128.8, 127.1, 126.6, 126.3, 119.0, 105.5, 55.3, 54.0, 18.3, 11.8.

HRMS: ESI, [M+H]^+^ Calcd. For (C_15_H_17_O_2_S): 261.0944. Found: 261.095.

[α]^25^_D_ = 65.6 (c = 0.5, CHCl_3_).

The compound exhibited identical ^1^H and ^13^C {^1^H} NMR data to previous reports.^14^

***S*-methyl 2-(4-(2,2-dichlorocyclopropyl)phenoxy)-2-methylpropanethioate (7f)**

Product **7f** was prepared according to the general procedure A (233 mg, 73% yield), and obtained as a

orange liquid.

^1^H NMR (500 MHz, CDCl_3_) δ 7.14 (d, *J* = 8.5 Hz, 2H), 6.93 (d, *J* = 8.6 Hz, 2H), 2.85 (dd, *J* = 10.4, 8.6 Hz, 1H), 2.31 (s, 3H), 1.95 (dd, *J* = 10.7, 7.4 Hz, 1H), 1.79 (t, *J* = 7.9 Hz, 1H), 1.53 (s, 6H).

^13^C {^1^H} NMR (126 MHz, CDCl_3_) δ 205.7, 153.9, 129.5, 129.2, 120.8, 85.7, 60.7, 34.8, 25.8, 25.5, 11.4.

HRMS: ESI, [M+H]^+^ Calcd. For (C_14_H_17_Cl_2_O_2_S): 319.0320. Found: 319.0323.

***S*-ethyl 2-(4-(2,2-dichlorocyclopropyl)phenoxy)-2-methylpropanethioate (7g)**

Product **7g** was prepared according to the general procedure A (292 mg, 88% yield), and obtained as a pale-yellow liquid.

^1^H NMR (500 MHz, CDCl_3_) δ 7.13 (d, *J* = 8.5 Hz, 2H), 6.92 (d, *J* = 8.5 Hz, 2H), 2.91 – 2.80 (m, 3H), 1.94 (dd, *J* = 10.7, 7.4 Hz, 1H), 1.78 (t, *J* = 7.9 Hz, 1H), 1.52 (s, 6H), 1.26 (t, *J* = 7.4 Hz, 3H).

^13^C {^1^H} NMR (126 MHz, CDCl_3_) δ 205.1, 153.9, 129.5, 129.1, 120.7, 85.6, 60.8, 34.8, 25.8, 25.4, 25.4, 22.9, 14.4.

HRMS: ESI, [M+H]^+^ Calcd. For (C_15_H_19_Cl_2_O_2_S): 333.0477. Found: 333.0491.

***S-*methyl 2-(1,3-dimethyl-2,6-dioxo-1,2,3,6-tetrahydro-9H-purin-9-yl)ethanethioate (7h)**

Product **7h** was prepared according to the general procedure A (228 mg, 85% yield), and obtained as a white powder solid. Pet ether/ethyl acetate (8.9:1.1) was used as a mobile phase for column chromatography.

Melting Point = 154-156 ^o^C.

^1^H NMR (500 MHz, CDCl_3_) δ 7.63 (s, 1H), 5.27 (s, 2H), 3.63 (s, 3H), 3.41 (s, 3H), 2.40 (s, 3H).

^13^C {^1^H} NMR (126 MHz, CDCl_3_) δ 193.5, 155.2, 151.6, 148.6, 142.0, 107.0, 54.3, 29.8, 27.9, 11.5.

HRMS: ESI, [M+H]^+^ Calcd. For (C_10_H_13_N_4_O_3_S): 269.0703. Found: 269.0712.

***S*-methyl 3-methyl-4-oxo-2-phenyl-4*H*-chromene-8-carbothioate (7i)**

Product **7i** was prepared according to the general procedure A (267 mg, 86% yield), and obtained as a light pink solid.

^1^H NMR (500 MHz, CDCl_3_) δ 8.45 (dd, *J* = 7.9, 1.3 Hz, 1H), 8.16 (dd, *J* = 7.5, 1.3 Hz, 1H), 7.77 (dd, *J* = 7.3, 1.9 Hz, 2H), 7.58- 7.51 (m, 3H), 7.46 (t, *J* = 7.7 Hz, 1H), 2.49 (s, 3H), 2.23 (s, 3H).

^13^C {^1^H} NMR (126 MHz, CDCl_3_) δ 189.6, 178.1, 161.1, 152.7, 133.5, 132.7, 130.4, 130.3, 129.4, 128.4, 127.7, 124.1, 123.1, 117.9, 12.6, 11.8.

The compound exhibited identical ^1^H and ^13^C {^1^H} NMR data to previous reports.^5^

***S*-methyl 4-(heptan-4-ylsulfonyl)benzothioate (7j)**

Product **7j** was prepared according to the general procedure A (252 mg, 80% yield), and obtained as a colorless oil.

^1^H NMR (500 MHz, CDCl_3_) δ 8.07 (d, *J* = 8.3 Hz, 2H), 7.89 (d, *J* = 8.3 Hz, 2H), 3.14-3.07 (m, 4H), 2.52 (s, 3H), 1.60-1.51 (m, 4H), 0.87 (t, *J* = 7.4 Hz, 6H).

^13^C {^1^H} NMR (126 MHz, CDCl_3_) δ 191.3, 144.3, 139.6, 127.6, 127.2, 49.9, 21.9, 11.9, 11.1.

HRMS: ESI, [M+H]^+^ Calcd. For (C_15_H_22_O_3_S_2_): 316.10356. Found: 316.1042. The compound exhibited identical ^1^H and ^13^C {^1^H} NMR data to previous reports.^5^

**4. References:**

1. Patel, M. A., Vora, R. K., Sanghvi, Y. S. & Kapdi, A. R. Ambient Temperature Metal-Free Thiomethylation of Chloroheteroarenes and Chloropurines. *Chem. – An Asian J.* **19**, e202400114 (2024).

2. Kristensen, S. K., Laursen, S. L. R., Taarning, E. & Skrydstrup, T. Ex Situ Formation of Methanethiol: Application in the Gold(I)-Promoted Anti-Markovnikov Hydrothiolation of Olefins. *Angew. Chemie - Int. Ed.* **57**, 13887–13891 (2018).

3. Vaidyanathan, R., Kalthod, V. G., Ngo, D. P., Manley, J. M. & Lapekas, S. P. Amidations Using N,N ‘-Carbonyldiimidazole: Remarkable Rate Enhancement by Carbon Dioxide. *J. Org. Chem.* **69**, 2565–2568 (2004).

4. Zhang, C. *et al.* Cesium carbonate-promoted synthesis of aryl methyl sulfides using: S -methylisothiourea sulfate under transition-metal-free conditions. *Org. Biomol. Chem.* **16**, 6316–6321 (2018).

5. Huang, D.-Y. *et al.* KF-catalyzed direct thiomethylation of carboxylic acids with DMSO to access methyl thioesters. *Org. Biomol. Chem.* **22**, 1453–1457 (2024).

6. Sharma, A. K. *et al.* Supported Palladium-Gold Catalyzed Carbonylative Methylthioesterification of Aryl Iodides using Oxalic acid and DMSO as CO and CH3SH Surrogates. *Asian J. Org. Chem.* **9**, 2099–2102 (2020).

7. Li, Y., Bao, G. & Wu, X. F. Palladium-catalyzed intermolecular transthioetherification of aryl halides with thioethers and thioesters. *Chem. Sci.* **11**, 2187–2192 (2020).

8. Mai, W.-P., Sui, H.-D., Lv, M.-X. & Lu, K. Nickel-catalyzed carbonylation of thioacetates with aryl iodides via CO insertion and C–S bond cleavage. *J. Chem. Res.* **45**, 890–895 (2021).

9. Qi, X., Bao, Z.-P., Yao, X.-T. & Wu, X.-F. Nickel-Catalyzed Thiocarbonylation of Arylboronic Acids with Sulfonyl Chlorides for the Synthesis of Thioesters. *Org. Lett.* **22**, 6671–6676 (2020).

10. Ueno, Y., Nozomi, M. & Okawara M. Direct Synthesis of Protected Thiols By Tributylstannyl Group. *Chem. Lett.* **11**, 1199–1202 (1982).

11. Mamane, V., Aubert, E. & Fort, Y. The Methyl Group as a Source of Structural Diversity in Heterocyclic Chemistry:  Side Chain Functionalization of Picolines and Related Heterocycles. *J. Org. Chem.* **72**, 7294–7300 (2007).

12. Barbero, M., Cadamuro, S., Degani, I., Dughera, S. & Fochi, R. Synthetic application of lithiated tris(methylthio)methane: preparation of aliphatic methyl thiolcarboxylates from the corresponding halides. Convenient synthesis of tris(methylthio)methane. *J. Chem. Soc. Perkin Trans. 1* 2075–2080 (1993) doi:10.1039/P19930002075.

13. Wehofsky, N., Koglin, N., Thust, S. & Bordusa, F. Reverse Proteolysis Promoted by in Situ Generated Peptide Ester Fragments. *J. Am. Chem. Soc.* **125**, 6126–6133 (2003).

14. Clericuzio Iacopo; Dughera, Stefano; Fochi, Rita, M. D. An Interesting Synthetic Application of S-Alkyl (Aryl)bis(alkylsulfanyl)thio­acetates: General Procedure for the Preparation of (±)-α-Arylpropionic Acids. *Synthesis (Stuttg).* **2002**, 921–927 (2002).

**5. ^1^H and ^13^C Spectral Data**

^1^H NMR spectrum of **3a** (500 MHz, CDCl_3_)

^13^C {^1^H} NMR spectrum of **3a** (126 MHz, CDCl_3_)

^1^H NMR spectrum of **3b** (500 MHz, CDCl_3_)

^13^C {^1^H} NMR spectrum of **3b** (126 MHz, CDCl_3_)

^1^H NMR spectrum of **3c** (500 MHz, CDCl_3_)

^13^C {^1^H} NMR spectrum of **3c** (126 MHz, CDCl_3_)

^1^H NMR spectrum of **3d** (500 MHz, CDCl_3_)

^13^C {^1^H} NMR spectrum of **3d** (126 MHz, CDCl_3_)

^1^H NMR spectrum of **3e** (500 MHz, CDCl_3_)

^13^C {^1^H} NMR spectrum of **3e** (126 MHz, CDCl_3_)

^1^H NMR spectrum of **3f** (500 MHz, CDCl_3_)

^13^C {^1^H} NMR spectrum of **3f** (126 MHz, CDCl_3_)

^1^H NMR spectrum of **3g** (500 MHz, CDCl_3_)

^13^C {^1^H} NMR spectrum of **3g** (126 MHz, CDCl_3_)

^1^H NMR spectrum of **3h** (400 MHz, CDCl_3_)

^13^C {^1^H} NMR spectrum of **3h** (101 MHz, CDCl_3_)

^1^H NMR spectrum of **3i** (400 MHz, CDCl_3_)

^13^C {^1^H} NMR spectrum of **3i** (101 MHz, CDCl_3_)

^1^H NMR spectrum of **3j** (400 MHz, CDCl_3_)

^13^C {^1^H} NMR spectrum of **3j** (101 MHz, CDCl_3_)

^1^H NMR spectrum of **3k** (500 MHz, CDCl_3_)

^13^C {^1^H} NMR spectrum of **3k** (126 MHz, CDCl_3_)

^1^H NMR spectrum of **3l** (500 MHz, CDCl_3_)

^13^C {^1^H} NMR spectrum of **3l** (126 MHz, CDCl_3_)

^1^H NMR spectrum of **3m** (500 MHz, CDCl_3_)

^13^C {^1^H} NMR spectrum of **3m** (126 MHz, CDCl_3_)

^1^H NMR spectrum of **3n** (500 MHz, CDCl_3_)

^13^C {^1^H} NMR spectrum of **3n** (126 MHz, CDCl_3_)

^1^H NMR spectrum of **3o** (500 MHz, CDCl_3_)

^13^C {^1^H} NMR spectrum of **3o** (126 MHz, CDCl_3_)

^1^H NMR spectrum of **3p** (500 MHz, CDCl_3_)

^13^C {^1^H} NMR spectrum of **3p** (126 MHz, CDCl_3_)

^1^H NMR spectrum of **3q** (500 MHz, CDCl_3_)

^13^C {^1^H} NMR spectrum of **3q** (126 MHz, CDCl_3_)

^1^H NMR spectrum of **3r** (500 MHz, CDCl_3_)

^13^C {^1^H} NMR spectrum of **3r** (126 MHz, CDCl_3_)

^1^H NMR spectrum of **3s** (500 MHz, CDCl_3_)

^13^C {^1^H} NMR spectrum of **3s** (126 MHz, CDCl_3_)

^1^H NMR spectrum of **3t** (500 MHz, CDCl_3_)

^13^C {^1^H} NMR spectrum of **3t** (126 MHz, CDCl_3_)

^1^H NMR spectrum of **3u** (500 MHz, CDCl_3_)

^13^C {^1^H} NMR spectrum of **3u** (126 MHz, CDCl_3_)

^1^H NMR spectrum of **3v** (500 MHz, CDCl_3_)

^13^C {^1^H} NMR spectrum of **3v** (126 MHz, CDCl_3_)

^1^H NMR spectrum of **5a** (500 MHz, CDCl_3_)

^13^C {^1^H} NMR spectrum of **5a** (126 MHz, CDCl_3_)

^1^H NMR spectrum of **5b** (500 MHz, CDCl_3_)

^13^C {^1^H} NMR spectrum of **5b** (126 MHz, CDCl_3_)

^1^H NMR spectrum of **5c** (500 MHz, CDCl_3_)

^13^C {^1^H} NMR spectrum of **5c** (126 MHz, CDCl_3_)

^1^H NMR spectrum of **5d** (500 MHz, CDCl_3_)

^13^C {^1^H} NMR spectrum of **5d** (126 MHz, CDCl_3_)

^1^H NMR spectrum of **5e** (500 MHz, CDCl_3_)

^13^C {^1^H} NMR spectrum of **5e** (126 MHz, CDCl_3_)

^1^H NMR spectrum of **5f** (500 MHz, CDCl_3_)

^13^C {^1^H} NMR spectrum of **5f** (126 MHz, CDCl_3_)

^1^H NMR spectrum of **7a** (500 MHz, CDCl_3_)

^13^C {^1^H} NMR spectrum of **7a** (126 MHz, CDCl_3_)

^1^H NMR spectrum of **7b** (500 MHz, CDCl_3_)

^13^C {^1^H} NMR spectrum of **7b** (126 MHz, CDCl_3_)

^1^H NMR spectrum of **7c** (500 MHz, CDCl_3_)

^13^C {^1^H} NMR spectrum of **7c** (126 MHz, CDCl_3_)

^1^H NMR spectrum of **7d** (500 MHz, CDCl_3_)

^13^C {^1^H} NMR spectrum of **7d** (126 MHz, CDCl_3_)

^1^H NMR spectrum of **7e** (500 MHz, CDCl_3_)

^13^C {^1^H} NMR spectrum of **7e** (126 MHz, CDCl_3_)

^1^H NMR spectrum of **7f** (500 MHz, CDCl_3_)

^13^C {^1^H} NMR spectrum of **7f** (126 MHz, CDCl_3_)

^1^H NMR spectrum of **7g** (500 MHz, CDCl_3_)

^13^C {^1^H} NMR spectrum of **7g** (126 MHz, CDCl_3_)

^1^H NMR spectrum of **7h** (500 MHz, CDCl_3_)

^13^C {^1^H} NMR spectrum of **7h** (126 MHz, CDCl_3_)

^1^H NMR spectrum of **7i** (500 MHz, CDCl_3_)

^13^C {^1^H} NMR spectrum of **7i** (126 MHz, CDCl_3_)

^1^H NMR spectrum of **7j** (500 MHz, CDCl_3_)

^13^C {^1^H} NMR spectrum of **7j** (126 MHz, CDCl_3_)

**6. X-ray Crystal data**

**6.1 Crystal data and structure refinement for 3d:**

(Ellipsoid is drawn at the 50% Probability level)

The single crystal of **3d** was prepared through recrystallization in mixture of solvent CHCl_3_ and CH_2_Cl_2_ in a vial by slow solvent evaporation at room temperature.


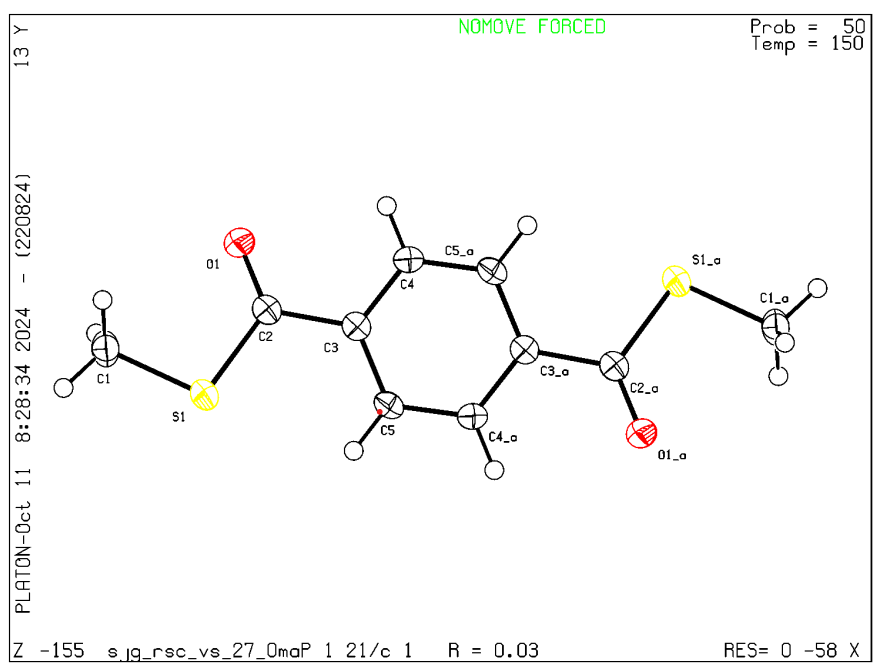


| Identification code |  | **3d** |  |
| --- | --- | --- | --- |
| Solvent |  | CH_2_Cl_2_ |  |
| CCDC | 2405141 |  |  |
| Bond precision: | C-C = 0.0020 A | Wavelength=0.71073 |  |
| Cell: | a= 9.2937(7) | b= 7.7624(5) | c= 7.8968(6) |
|  | alpha=90 | beta= =114.166(3) | gamma=90 |
| Temperature: | 150 K |  |  |
|  | Calculated | Reported |  |
| Volume | 519.76(7) | 519.76(7) |  |
| Space group | P 21/c | P 1 21/c 1 |  |
| Hall group | -P 2ybc | -P 2ybc |  |
| Moiety formula | C10 H10 O2 S2 | C10 H10 O2 S2 |  |
| Sum formula | C10 H10 O2 S2 | C10 H10 O2 S2 |  |
| Mr | 226.30 | 226.30 |  |
| Dx, g cm-3 | 1.446 | 1.446 |  |
| Z | 2 | 2 |  |
| Mu (mm-1) | 0.481 | 0.481 |  |
| F000 | 236.0 | 236.0 |  |
| F000’ | 236.59 |  |  |
| h,k,l max | 11, 9, 9 | 11, 9, 9 |  |
| Nref | 924 | 918 |  |
| Tmin,Tmax | 0.895, 0.923 | 0.307, 0.461 |  |
| Tmin’ | 0.861 |  |  |
| Correction method =  AbsCorr = NUMERICAL | # Reported T | Limits: Tmin=0.307 | Tmax=0.461 |
| Data completeness = | 0.994 | Theta(max)= 25.054 |  |
| R(reflections) = | 0.0258( 871) | wR2(reflections)= 0.0678( 918) |  |
| S = 1.122 | Npar = 65 |  |  |

**6.2 Crystal data and structure refinement for 5e:**

**X-Ray crystallographic analysis: Crystal data and structure refinement for 5e**

(Ellipsoid is drawn at the 50% Probability level)

The single crystal of **5e** was prepared through recrystallization in mixture of solvent CHCl_3_ and CH_2_Cl_2_ in a vial by slow solvent evaporation at room temperature.

**
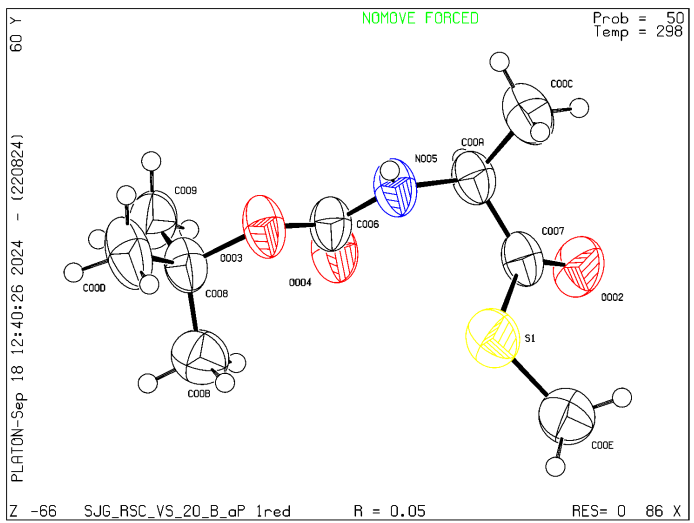
**

| Identification code |  | **5e** |  |
| --- | --- | --- | --- |
| Solvent |  | CH_2_Cl_2_ |  |
| CCDC | 2405142 |  |  |
| Bond precision: | C-C = 0.0042 A | Wavelength=0.71073 |  |
| Cell: | a=5.2644 (5) | b=6.0763 (5) | c=10.3890 (8) |
|  | alpha=102.963(7) | beta=103.114(7) | gamma=100.007(7) |
| Temperature: | 298 K |  |  |
|  | Calculated | Reported |  |
| Volume | 306.45(5) | 306.44(5) |  |
| Space group | P 1 | P 1 |  |
| Hall group | P 1 | P 1 |  |
| Moiety formula | C9 H17 N O3 S | C9 H17 N O3 S |  |
| Sum formula | C9 H17 N O3 S | C9 H17 N O3 S |  |
| Mr | 219.30 | 172.21 |  |
| Dx, g cm-3 | 1.188 | 0.933 |  |
| Z | 1 | 1 |  |
| Mu (mm-1) | 0.249 | 0.060 |  |
| F000 | 118.0 | 91.1 |  |
| F000’ | 118.18 |  |  |
| h,k,l max | 8, 10, 17 | 8, 9, 17 |  |
| Nref | 5874[ 2937] | 4179 |  |
| Tmin,Tmax | 0.976, 0.981 | 0.786, 1.000 |  |
| Tmin’ | 0.960 |  |  |
| Correction method =  AbsCorr = MULTI-SCAN | # Reported T | Limits: Tmin=0.786 | Tmax=1.000 |
| Data completeness = | 1.42/0.71 | Theta(max)= 36.130 |  |
| R(reflections) = | 0.0531( 2287) | wR2(reflections)= 0.1713( 4179) |  |
| S = 0.881 | Npar = 132 |  |  |

**7. Controlled experiment NMR spectra:**

^1^H and ^13^C NMR of **Benzoic acid**

^1^H and ^13^C NMR of **A**: After 0.5 h (intermediate formation)

^1^H NMR of **B**: After 1.0 h (intermediate formation)

^1^H NMR of **C**: After 1.5 h (After gas generation in chamber 2)

^1^H and ^13^C NMR of **D**: After 3.0 h (After gas generation in chamber 2)

^1^H NMR spectrum Benzoic acid (400 MHz, CD_3_CN)

^13^C {^1^H} NMR spectrum of Benzoic acid (101 MHz, CD_3_CN)

^1^H NMR spectrum A (400 MHz, CD_3_CN)

^1^H NMR spectrum A (400 MHz, CD_3_CN)

^13^C {^1^H} NMR spectrum of **A** (101 MHz, CD_3_CN)

^13^C {^1^H} NMR spectrum of **A** (101 MHz, CD_3_CN)

^1^H NMR spectrum **B** (400 MHz, CD_3_CN)

^1^H NMR spectrum **B** (400 MHz, CD_3_CN)

^1^H NMR spectrum **C** (400 MHz, CD_3_CN)

^1^H NMR spectrum **C** (400 MHz, CD_3_CN)

^1^H NMR spectrum **D** (400 MHz, CD_3_CN)

^1^H NMR spectrum **D** (400 MHz, CD_3_CN)

^13^C {^1^H} NMR spectrum of **D** (101 MHz, CD_3_CN)

^13^C {^1^H} NMR spectrum of **D** (101 MHz, CD_3_CN)
